# Scaffolded acquisition of a robot self–world distinction in a layered architecture model of the developing self

**DOI:** 10.1098/rstb.2025.0153

**Published:** 2026-11-10

**Authors:** Luca Manneschi, Alejandro Jimenez-Rodriguez, Daniel Camilleri, Tony J. Prescott

**Affiliations:** ^1^ School of Computer Science, The University of Sheffield, Sheffield, UK; ^2^ Department of Computing, Sheffield Hallam University, Sheffield, UK; ^3^ Kinematic Trees Ltd, Sheffield, UK

**Keywords:** cognitive neurorobotics, embodied cognition, anoetic self, sense of ownership, sense of agency, latent representation, neural ODEs, multisensory integration, superior colliculus, parietal cortex

## Abstract

How does the sense of self emerge in human development, and could an artificial system acquire a similar sense using algorithmic principles and given a suitable embodiment and experience? Here, we describe a machine learning framework, inspired by developmental psychology and neurobiology, that acquires a self–world distinction and minimal self-model. Unlike previous approaches, we introduce a gradual formulation, integrating correlation-based and predictive techniques within a layered architecture. Using a simulated humanoid, we show first how a self–world distinction can emerge through correlation-based self-supervision. This model uses dependencies between proprioception and vision to distinguish its own body from its surroundings during infant-like motor babbling. Next, we construct a model of how these sensory signals will evolve in time. This predictive capacity uses the self–world distinction acquired by the lower layer as input to neural ordinary differential equation models that learn continuous dynamics. Finally, we demonstrate scaffolding of the higher layer by the lower one, showing that predictive learning occurs more rapidly with the lower layer already in place. By integrating these mechanisms, our approach offers a framework for characterizing a minimal robotic self that can inform our understanding of the emergence of the sense of self in the human mind/brain.

This article is part of the discussion meeting issue ‘From being to identity: analysis and synthesis of the self’.

## 1. Introduction

The *self–world distinction* is the separation of experience (perceptual and cognitive) as deriving from the self (‘me’) or from its counterpart, the world (‘not me’) [1]. For the human self, this distinction seems closely tied to the *sense of ownership* of the physical body and of signals emanating from inside body (or as relating to the body, for instance, when you observe yourself) and to the *sense of agency*—the experience of owning and originating actions and their consequences [2]. Following Piaget [3], the distinction between self and world may also be seen as a prerequisite for a *self–other distinction* with respect to the social world [4,5], that is, the separation of the mental life of the self from that of other selves [1,6]. Although conceptually we can distinguish these different aspects of the sense of self, understanding how they stand with respect to each other and how each might emerge in both evolution and development remains mysterious. Here, we take a synthetic ‘understanding through building’ approach [7–9] to begin to untangle these interdependencies.

As we will explore in this article, the self–world distinction is not all-or-nothing and can manifest in different ways. It can be non-conceptual, or *anoetic*, where the self is experienced as distinct but without any insight or self-awareness [10,11]. On the other hand, it can also be reflective, or *noetic*, where the self can be a subject of thought and judgement [10,12]. Finally, it can be *autonoetic* where the self can be conceived of and experienced as existing across time [12–14]. These three aspects can be present alongside each other. The anoetic aspects may contribute a feeling of the self as motivated, integrated and bounded, while the noetic aspects become more prominent during self-reflection and autonoetic when remembering the past or imagining the future.

In psychiatric conditions, different aspects of the self–world distinction can become disturbed, such as in *asomatognosia* where a part of the body is experienced as not belonging to the self, or in *autoscopia* where a visual hallucination of the self is projected into the outside world [15]. Likewise, altered states of consciousness, such as those induced by meditation or psychoactive drugs, can lead to a partial dissolution of the self–world boundary [16–18]. Experimental devices, such as the rubber hand illusion, and technologies, such as virtual reality and robotic telepresence, can also alter the experience of the self–world boundary, such that we might experience ownership of an artificial hand [19] or have a feeling of the self as being outside the physical body [20,21]. This evidence points to the actively and continuously constructed nature of the experience of selfhood [22].

In developmental terms, construction of a self–world distinction likely begins with the infant's own body and with the interoceptive, proprioceptive and somatosensory signals it generates [5]. For instance, basic visceral neural circuits, such as those underlying heartbeat, breathing and swallowing, emerge by the second trimester of pregnancy [23] and could provide a backdrop of predictable internal sensory dynamics to which new kinds of signals can be associated as also belonging to the self [14,24]. Proprioceptive signals provide evidence to the developing brain of its own agency as they vary in correlation with internally generated motor signals [5,25,26]. Somatosensory signals emanating from the skin—the body–world boundary—afford different experiences when the unborn baby touches innervated and non-innervated regions of her body [27]. This may provide the developing fetus with one of the earliest indications of her separate and bounded nature.

By the time we are born, human experience likely includes a distinction between self and world, albeit of an anoetic nature [5,27,28]. This is indicated, for instance, by a touch on the cheek of a neonate which will trigger a rooting response except when the infant touches herself [29]. In the newborn, hearing and vision offer new means of learning about the self through *reafference*—observation of the sensory consequences of self-generated movements and sounds [5,30,31]. For instance, when awake and alert, and in a supported position, a newborn may engage in arm and hand movements, a portion of which will fall within her visual field [32]. These movements, which appear exploratory but not goal-directed and are sometimes referred to as hand or motor ‘babbling’ [33,34], provide a new means of distinguishing the bodily self and its actions. By three months, exploratory movements are largely replaced by coordinated visually guided reaching that appears more intentional [35] and, by around six months, by reach and grasp behaviours that are suitably adapted to context [36,37].

In this contribution, we are specifically concerned with the question of how reafference might contribute to the emergence of the self–world distinction. We take a synthetic approach [7,9] using machine learning and (simulated) robotic modelling to explore how visual reafference, as in the infant's observations of her own limb movements, might guide the emergence of a visual self–world distinction (VSWD). We have previously argued that a unified experience of self emerges from the activity of multiple self ‘subsystems’ in the mind/brain [8,22,38]. Moreover, building on early proposals from Damasio [39], Panksepp [11,40] and Northoff *et al.* [41], we have also argued that this ‘self system’ is organized in the form of a layered architecture [42,43], whereby some core aspects of self—principally those related to anoetic senses of agency and ownership—are available in young infants (0–3 months of age), while other aspects emerge more gradually during childhood [14,22]. For instance, while the newborn is thought to live ‘in the moment’, by age two, the developing child will begin to distinguish past from future. However, this is still only the beginning of the emergence of a sense of the self in time. Full autonoetic experience, including the capacity to mentally ‘time travel’ into the past or future, may only arise alongside the acquisition of autobiographical memory capacities by age 4 or later [13,14,44].

According to this view, postnatal experience, alongside brain maturation, is critical in assembling the human sense of self. Here, we propose and test, using a robot simulation of motor babbling, a layered architecture hypothesis for the acquisition of a VSWD during the first few months of infancy. Specifically, as illustrated in figure 1, we first model a learning layer, inspired by Hebbian principles, that uses the correlation between visual and proprioceptive signals to provide an initial decomposition of the visual world into self and non-self components. We then extend this model with a predictive learning layer that constructs a dynamical model of the relationship between proprioception and visual experience of the self and is able to predict how each could evolve in the near future. We show that by training the simpler correlation-learning layer first, and by providing its *latent* (internal) representation of self–world as input to the predictive layer, we are able to ‘scaffold’ [43,46] the capacity of the latter to learn effectively and quickly.

**Figure 1.**
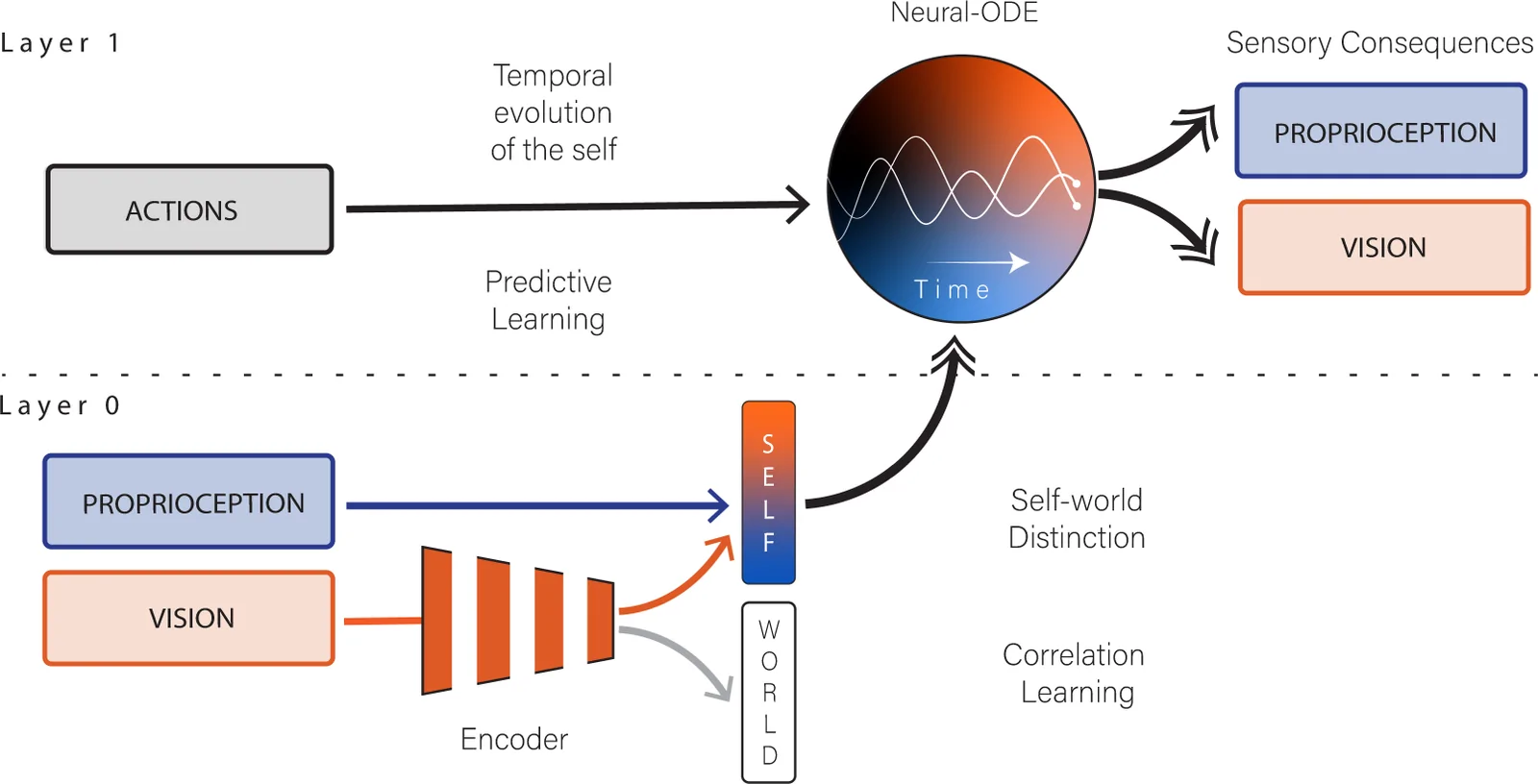
A layered architecture for the self–world distinction. Layer 0 acquires the ability to segregate sensory signals generated by self and world through correlation-based learning between proprioceptive and visual signals. This provides an anoetic sense of ownership over parts of the visual field occupied to the body. Layer 1, which becomes active once layer 0 training has taken place, adds a predictive capability that anticipates how both visual and proprioceptive signals will evolve in time. Sense of agency can be characterized as the experience of congruence between the predicted sensory consequences of action and the actual consequences [26,45]. Since layer 1 generates exactly these kinds of predictions, it could be viewed as providing the foundations for an anoetic sense of agency (see Discussion).

As further set out below, both learning systems are forms of deep neural network building on current approaches in machine learning. To be clear, we are not arguing that these are strongly analogous to specific brain processes since we are not modelling brain circuits directly or attempting to closely match the visual and proprioceptive systems of the infant. Nevertheless, we do consider that this work can be informative in understanding the organization and emergence of the self–world distinction in the human mind/brain and return to this topic in the discussion in relation to potential cortical and subcortical substrates for distinguishing self from non-self. We next describe our robot simulation environment and the design and testing of, first, the correlation learning model and, second, the full model with correlation-based and predictive learning layers.

## 2. Layered architecture model

### (a) Humanoid robot simulation

We use a full-physics simulation of the Unitree G1 humanoid (figure 2) using a faster than real-time GPU (graphics processing unit)-accelerated simulation, ManiSkill [47]. We select the G1 as it has a human-like morphology, including two legs, two arms and two three-fingered hands. The robot's 23 degrees of freedom allow it to make movements of the arms and legs that resemble those of humans, although it has no neck joints. The robot is equipped with a head-mounted monocular RGB-D (red, green, blue–depth) camera with segmentation, with $85.2^{\circ }\times 58^{\circ }\times 94^{\circ }$ field of view. We use only the monocular two-dimensional RGB images for our study since infants’ eyes are poorly coordinated and stereo vision does not emerge until three to four months [48]. We place the robot on its back to resemble a human infant in a cot and add to the visible environment a collection of coloured boxes arranged in a rotating circle that resembles a baby ‘mobile’ (a patterned toy often suspended above a baby's cot that rotates to provide enriched visual experience).

**Figure 2.**
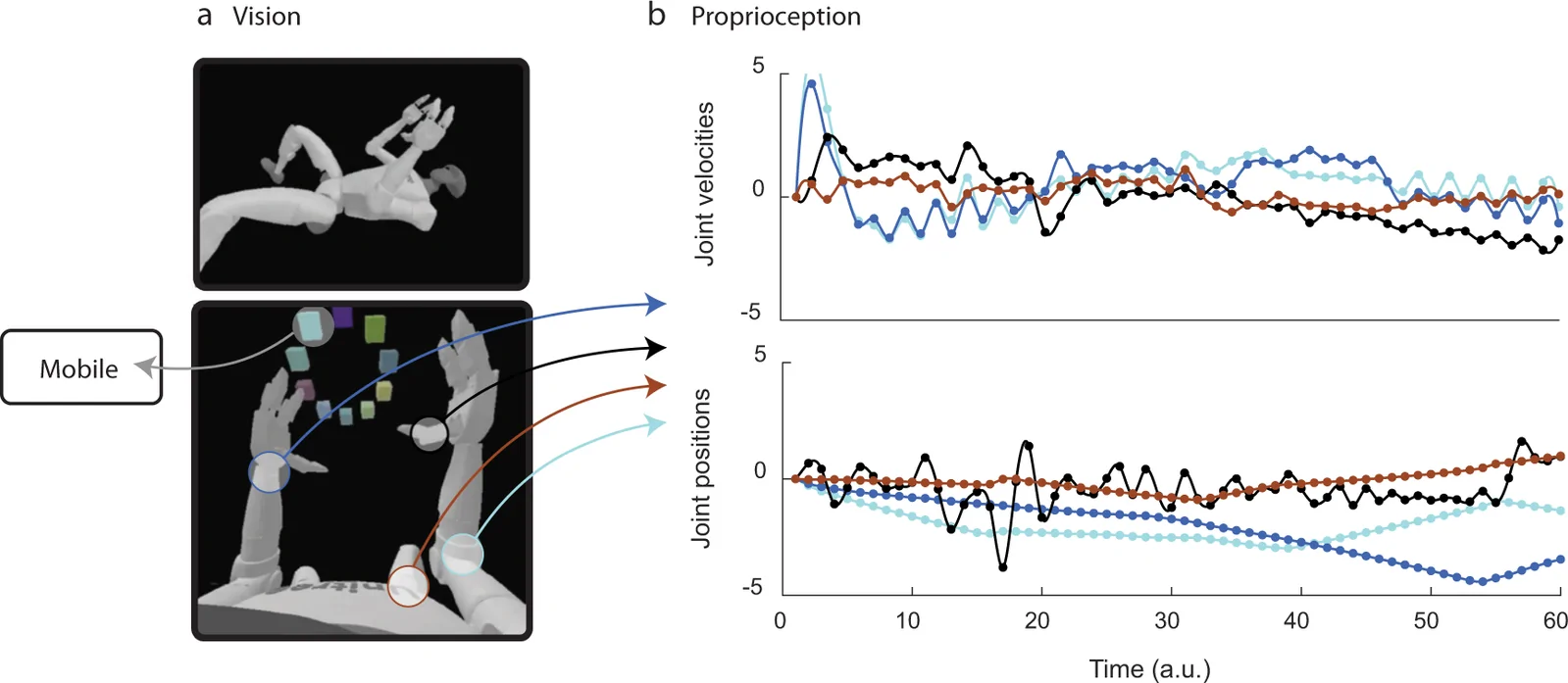
Illustration of the simulation set-up and data collection for the motor babbling task. (a) The simulated humanoid robot lies on its back (top view) observing its body, arms and hands through an RGB camera (bottom view) located in the head. We place a revolving visual pattern resembling a baby mobile in the robot's visual field against an otherwise black background. The mobile provides an external, moving object in the scene, which is not part of the robot, and can serve as a target for world segmentation. RGB images of 128 x 128 resolution are obtained at 30 fps. (b) Example proprioceptive data. Proprioceptive signals from 23 joints are collected at 20 Hz; the plots show example of how these evolve over time.

To generate training data for our models, we implement motor babbling by sampling target hand positions using a three-dimensional Gaussian with an assigned variance per dimension. Targets are represented as the desired change in position, Δ(x,y,z), with respect to the robot base, for each of the end-effectors (both hands). When a new target is chosen, we actuate the robot using Manikskill's built-in PID (proportional, integral, and derivative) controller, which issues expected delta movements to the robot's joints. This is done per end-effector, i.e. each hand samples from its own Gaussian distribution of target positions and is appropriately actuated to move towards it. Once the target position is reached, a new one is selected. The simulation is configured to run at 100 Hz (physics time step). Every five simulation steps (i.e. at a frequency of 20 Hz), the control loop receives sensory data—proprioceptive data (positions, velocities and effort per joint) and camera frames specified as ‘rgb+depth+segmentation’ with a resolution of 128 ×  128—and issues control commands. The depth map is ignored, while the segmentation image, which provides object identities (ids) per pixel, is further processed to create a self-segmentation ground truth where all the ids related to the robot's links are given an id of 1. Additionally, for each processed frame, we compute a scalar per-pixel estimate of optic flow to provide a motion cue.

As will become clear in the following sections, our layered architecture learns to disentangle *self* and *world* in its internal representation via self-supervised learning, while also predicting the consequences of its actions. To assess the quality of the resulting latent spaces, and to guide optimization, we adopt decoders that reconstruct the input modalities, making explicit what information is captured by each latent. Ideally, the self latent should reconstruct the body, whereas the world latent should reconstruct the external object (the mobile). For visual reconstructions, we quantify performance using *pixel-wise accuracy* and *soft Dice coefficient* [49] measures computed over a region of interest (ROI). Full definitions are provided in the supplementary material; here, we summarize the essential information. Given a decoded attention map, we retain the top-k attended pixels and compare them against the corresponding ground-truth segmentation. Pixel-wise accuracy is computed after hard-thresholding the reconstruction: we retain the top-k pixels, binarize them and compare the resulting mask to the ground-truth segmentation. By contrast, the soft Dice coefficient is computed on the continuous reconstructed values within the ROI, yielding a graded measure of overlap between prediction and ground truth without binarization. We will report pixel-wise accuracy when assessing the behaviour of the proposed architecture and illustrating basic qualitative trends as it has a simple and intuitive interpretation. The soft Dice coefficient is widely used in the development of image segmentation models as it provides a graded measure that can capture finer-grained overlap information; it is therefore useful for comparing *between* alternative training methods. We use it here when comparing different model variants in §2d and in the supplementary material.

### (b) Correlation-learning layer

#### (i) Overview

The correlation-learning layer, illustrated in figure 3a, is designed to differentiate between self and non-self, while also constructing a latent representation that can support higher-level components. We employ an encoder–decoder structure where the encoder is designed to separate self-related and world-related information into two distinct latent spaces. These representations enable the decoders to independently reconstruct both the agent (self) and its surrounding environment.

**Figure 3.**
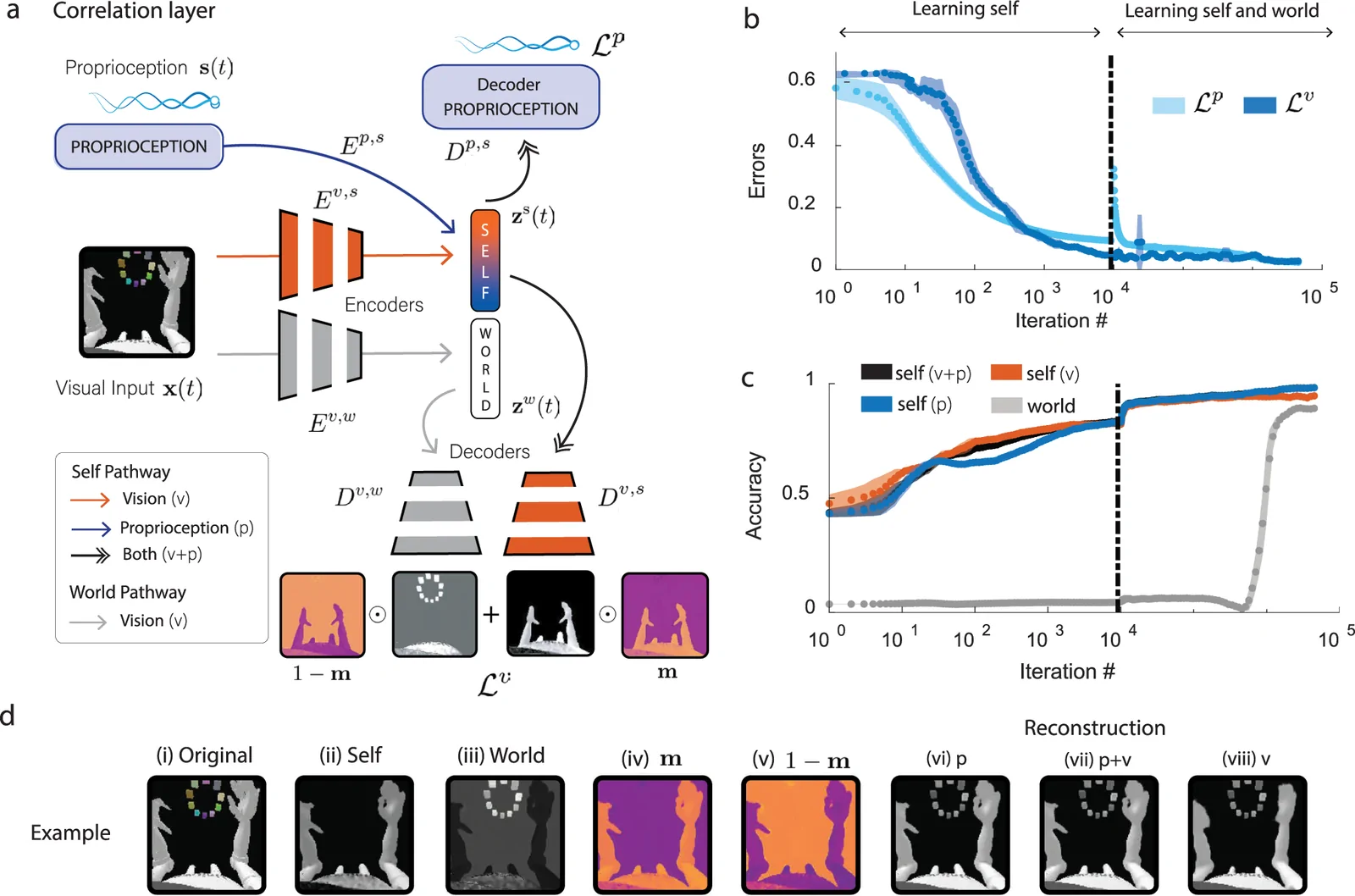
The correlation-learning layer. (a) Network architecture, where we adopt different latent spaces for the self and world information. (b) Learning curves for proprioceptive ($L^{p}$) and visual ($L^{v}$) reconstruction losses. The optimization proceeds in two stages, separated by the vertical dashed line at $10^{4}$ iterations. In the first stage, the world pathway is suppressed and the self pathway acquires a basic self representation; in the second stage, both pathways are active, and the network shifts to jointly learning the self and the world. (c) Pixel-level segmentation accuracy corresponding to the input configurations *proprioception only (p)*, *vision only (v)*, *proprioception and vision combined (p+v)* and *world*. The curves are smoothed using a running average, and the shaded bands denote the running standard deviation, providing an indication of training stability. The *x*-axis follows a piece-wise log-scale to better visualize the two training phases. (d). Example reconstructions. From left to right: (i) the original image; (ii) the decoded image from the *self* latent, $D^{v,s}(z^{s})$; (iii) the decoded image from the *world* latent, $D^{v,w}(z^{w})$; (iv)–(v) the self and world spatial masks (lighter colours indicate higher attention); and (vi)–(viii) full reconstructions under three input conditions: *proprioception only (p)*, *combined model (p+v)* and *vision only (v)*. See text for further explanation.

To reinforce the separation of self and world, the visual features contributing to the self-representation are passed through an information bottleneck, promoting a focus on self-related content. This can be understood as follows. Following the self pathway (black and red arrows in figure 3a), the latent representation associated with the self is used to reconstruct both proprioceptive and visual information. By contrast, the world-related pathway (grey arrows) is dedicated solely to reconstructing visual input. The demand for proprioceptive reconstruction in the self pathway acts as the bottleneck, effectively filtering for visual features that are specifically relevant to the self. Optimization proceeds in two stages. In stage 1 ($10^{4}$ iterations), we encourage the model to form a minimal sense of self by suppressing the world pathway. This forces the self pathway to learn an initial *spatial mask*, in the form of a 128 × 128 image filter with values in the range 0–1 per pixel, that will guide subsequent learning. In stage 2 (up to $10^{5}$ iterations), we train all pathways jointly. The significance of this sequential training process will be further analysed in §2d.

Overall, this network can be regarded as a correlation-learning mechanism because the encoder–decoder design compresses the inputs into a latent space that must capture statistical dependencies, including cross-modal correlations, in order to accurately reconstruct the information at the current time. As such, future observations are not required; the model operates solely on information available at the present time and does not perform any explicit prediction. The current model processes visual and proprioceptive data; however, the architecture is inherently modular and could be extended to integrate additional sensory signals, such as audition or touch.

#### (ii) Methods

We next explain the network architecture in more detail. As shown in figure 3a, we adopt separate visual encoders and decoders for the self and the world to prevent information leakage between the two processing pathways. Each encoder/decoder is implemented as a convolutional neural network, while the decoder for proprioceptive information is a multi-layer perceptron (MLP). We denote the visual input and its reconstruction by $x^{v}(t)$ and ${\hat{x}}^{v}(t)$, respectively, where both are 128×128×4 tensors of per-pixel RGB and optical-flow values. Similarly, we write the proprioceptive input and its reconstruction as s(t) and $\hat{s}(t)$, respectively, where both are 46-dimensional vectors of joint positions and velocities. The self and world latent spaces are $z^{s}(t)$ and $z^{w}(t)$. Finally, we denote the visual self-, visual world- and proprioceptive encoders by $E_{\phi^{v,s}}^{v,s}$, $E_{\phi^{\;v,w}}^{v,w}$ and $E_{\phi^{\;p,s}}^{p,s}$, respectively, and the corresponding decoders as $D_{\theta^{\;v,s}}^{v,s}$, $D_{\theta^{\;v,w}}^{v,w}$ and $D_{\theta^{\;p,s}}^{p,s}$. For notational convenience, we omit the lowercase parameter indices of the encoder and decoder architectures from now on and implicitly assume that each function is defined by its own set of parameters.

Under this notation, the latent spaces are defined as

(2.1)zs(t)=αEv,sxv(t)+(1−α)Ep,ss(t)(2.2)and zw(t)=Ev,wxv(t),

where α∈[0,1] balances the contributions of the visual and proprioceptive signals to the self latent and is randomly and uniformly sampled once per training sequence to promote robustness to changes in sensory-modality availability.

The proprioceptive reconstruction is $\hat{s}(t)=D^{p,s}\left(z^{s}(t)\right)$. For visual reconstruction, we combine the self and world decoders using a spatial attention mask $m(t)\in [0,1{]}^{H\times W}$ (broadcast along channels):

(2.3) $$\begin{array}{r} {\hat{x}}^{v}(t)=m(t)⊙D^{v,s}\left(z^{s}(t)\right)+\left(1-m(t)\right)⊙D^{v,w}\left(z^{w}(t)\right), \end{array}$$

where ⊙ denotes the element-wise (Hadamard) multiplication. The spatial attention mask $m(t)\in [0,1{]}^{H\times W}$ is computed from the decoded self-visual prediction as

(2.4) $$\begin{array}{r} m(t)\;=\;f\;\left({\left\langle D^{v,s}\;\left(z^{s}(t)\right)\right\rangle }_{\text{ch}}-c\right), \end{array}$$

i.e. we first take a channel-wise average $\langle \cdot \rangle_{\text{ch}}$ to obtain a single H×W intensity map and then apply a shifted sigmoid f(⋅) (with shift c) to obtain values in [0,1]. The constant c controls the operating region of the sigmoid (in practice, any c∈[0,1] works well, and we set c=0 in all simulations). Finally, the term 1−m(t) is the element-wise complement of m(t) and weights the world-related reconstruction accordingly.

Optimization of the layer is performed through backpropagation by minimizing the cost function:

(2.5) $$\begin{array}{r} L_{corr}=\|x^{v}(t)-{\hat{x}}^{v}(t){\|}_{1}+||s(t)-\hat{s}(t)|{|}_{2}^{2}+\beta \sum_{i=0}^{T-1}\|z^{s}(t_{i})-z^{s}(t_{i+1}){\|}_{2}^{2}=L^{v}+L^{p}+\beta \sum_{i=0}^{T-1}\|z^{s}(t_{i})-z^{s}(t_{i+1}){\|}_{2}^{2}, \end{array}$$

where $\|\cdot {\|}_{1}$ and $\|\cdot {\|}_{2}$ denote the mean absolute error and mean squared error, respectively, $t_{i}=t_{0}+i\Delta t$ and β is a scaling factor of the temporal regularization term. This third factor in the loss function enforces temporal proximity across time in the latent space and preparing $z^{s}$ to be modelled as a dynamical system (§2c). As noted above, there are two optimization stages. In stage 1, we set $z^{w}(t)\equiv 0$ to completely suppress the world pathway; we then remove this constraint in stage 2. For further details regarding the architecture and specific hyperparameters, see the supplementary material.

#### (iii) Results

Figure 3b,c shows training curves for the layer 0 model, generated using the data described in §2a, while figure 3d provides examples of the visual data, the computed separation of self and world and reconstructed images for different input configurations. Each of these can be understood as follows.

Figure 3b reports the proprioceptive ($L^{p}$) and visual ($L^{v}$) reconstruction errors calculated on the test set as learning progresses (training iteration number on the *x*-axis). These show that errors in both domains decrease smoothly during stage 1, with a brief uptick at the transition to stage 2 when the world pathway becomes active and optimization enters a new regime.

Figure 3c shows pixel-level self/world segmentation accuracy, computed by matching the reconstructed self and world images to ground-truth segmentations obtained *a priori* for self and world, respectively (§2a). We plot three self-accuracy curves corresponding to testing the trained system with different input configurations: *proprioception only (p)*, *vision only (v)* and a *combination of the two (p+v)*; we also plot one curve corresponding to reconstruction of the visual *world*. These metrics are computed by evaluating the model on a test set at logarithmically increasing intervals and using ground-truth segmentation provided by the simulator (§2a). The plots show that, in stage 1, the self pathway learns a coarse segmentation of its own body against the environment, while the world pathway remains at chance level (since its output is suppressed). After the transition to stage 2, self accuracy exhibits a sharp jump and continues to improve, and the world pathway learns to focus on objects in the scene (the mobile), achieving strong final performance. A coherent sense of self should be resilient to the loss of a modality; this is demonstrated by the relatively small differences between the various *self* curves—all testing regimes perform well, though the small drop in the *no-proprioception (v)* condition visible at the end of training underlines the importance of proprioceptive input for an optimal self representation. We further notice that performance in the proprioception only (*p*) condition is comparable to the one attained in the combined (*p+v*) setting. The reason is that proprioception acts as an explicit information bottleneck used to induce a self–world distinction. Because the self latent is trained to reconstruct proprioceptive signals, the model learns a proprioception-centred representation and naturally relies on proprioceptive inputs whenever they are available. Visual input can still contribute, but it becomes most relevant when proprioception is weak or absent, where it provides robustness by allowing the self-state to be inferred from visual cues alone.

Finally, figure 3d provides qualitative examples of the capacity of the model to reconstruct the visual world and to decompose it into self and world components. As expected, the image reconstructed by the self decoder (d*ii*) concentrates on the agent's body, whereas the world decoder highlights objects in the scene (d*iii*), specifically, the mobile. Notably, the world representation exhibits lower luminance over the arms and hands, while retaining some information about the more static torso. This is consistent with the torso's limited motion and the fact that static, low-frequency information is easier to reconstruct, enabling the world pathway to represent some torso-related information despite primarily focusing on external objects. The masks (d*iv* and d*v*) partition the scene appropriately, and the three rightmost reconstructions (d*vi*–d*viii*) illustrate that all input configurations yield accurate results, with the combined (*v+p*) and proprioception-only (*p*) conditions performing best.

### (c) Predictive-learning layer

#### (i) Overview

Beyond its role in generating a minimal VSWD, the correlation layer also supports the operation of the layer 1 predictive learning system. Specifically, its design encourages the emergence of a smoothly varying latent space that can be modelled as a dynamical system. After training, as described above, the weights in the correlation-learning layer are fixed. The predictive layer then builds on this system to provide a near-term forecast of what will happen next.

More specifically, the predictive layer is implemented as a neural ordinary differential equation (neural-ODE), i.e. an ordinary differential equation parametrized by a neural network. Rather than predicting the next system state directly, this network outputs the temporal derivative of the system state, and a numerical integrator is then used to obtain its evolution over time. The predictive layer thus allows us to evolve the latent state forward as a dynamical system driven by the planned action sequence. As a result, the model can imagine future latent states and, via the decoders, the corresponding future proprioceptive and visual observations. For comparison purposes, we also test a version in which the output of the model is provided as its own input in the next prediction step without a numerical integration procedure. This does not directly emulate a dynamical system but could generate similar outcomes. Optimization of this architecture proceeds with the *encoder* and *decoder* parameters kept frozen (unless otherwise specified in the experiments in§2d). This design lets the correlation layer scaffold the predictive layer and is also computationally efficient as explored further below.

To train the predictive model, we drive it with a sequence of external control signals corresponding to the relative change in hand position required to move to the next target position (as specified by the motor babbling); the outputs of the model are generated through the proprioceptive and visual decoders of the correlation layer for predictions of up to 100 time steps in the future.

#### (ii) Methods

As illustrated in figure 4a, the neural-ODE is defined over the self latent space $z^{s}(t)$ and is driven by an external control signal a(t), corresponding to the actions the robot intends to perform. The purpose of this layer is to forecast the future latent representation and associated proprioceptive states, given an initial condition $z^{s}(t_{0})$ and a sequence of actions a(t). For the current model, the sequence of actions is the sequence of target hand positions (§2a).

**Figure 4.**
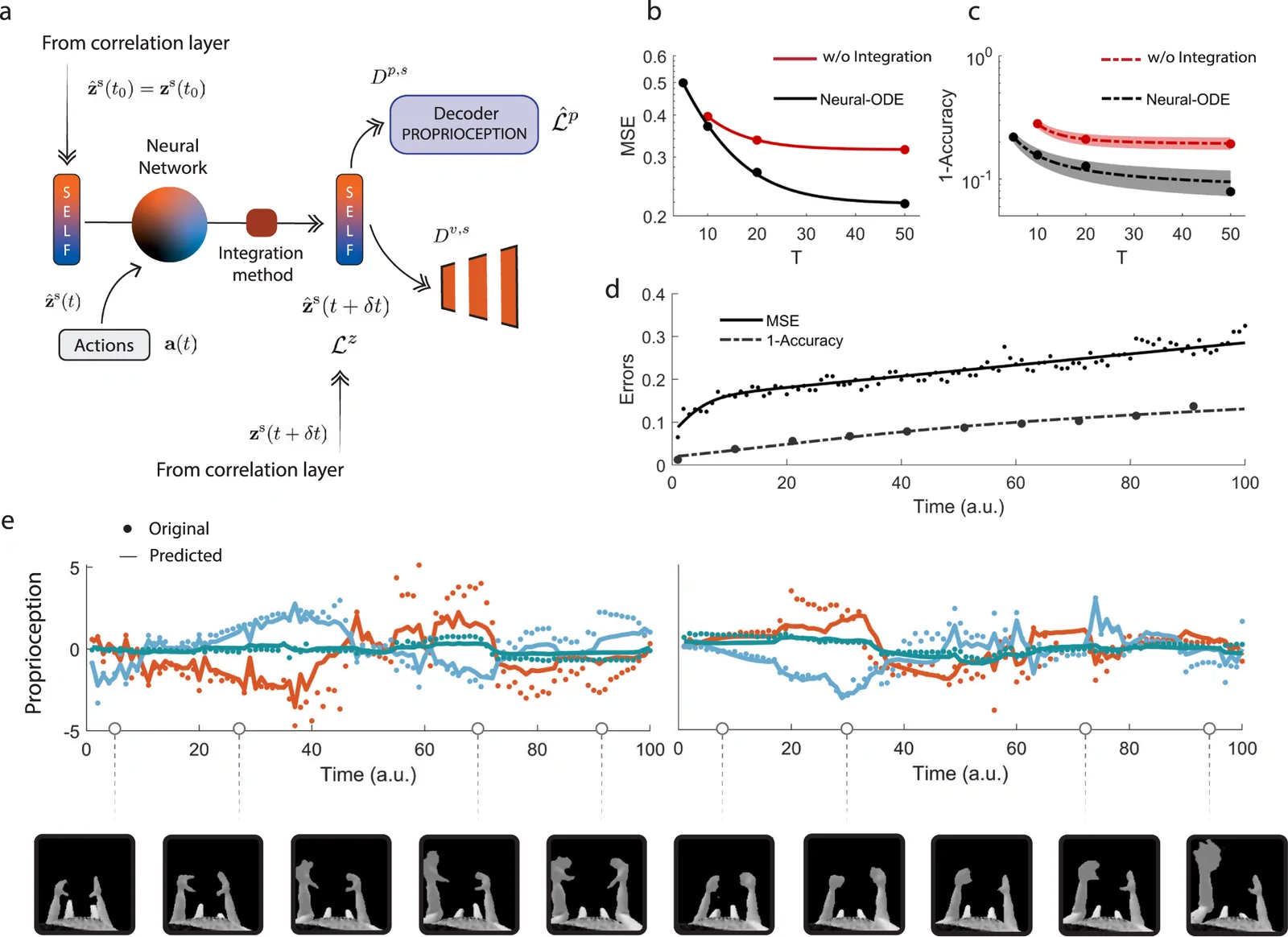
The prediction-learning layer. (a) Network architecture. (b) Mean squared error (MSE) of proprioceptive reconstructions for the neural-ODE and for an MLP trained without an explicit numerical integration scheme, as a function of the training sequence length (T). (c) As in (b), but reporting pixel-level error rate (1-accuracy). (d) Pixel-level accuracy and proprioceptive reconstruction error (MSE) as a function of prediction horizon for a neural-ODE model, with explicit integration, trained with T=50. The plot illustrates the accumulation of error as the predictive layer rolls the latent state further into the future (time on the *x*-axis). (e) Two examples (left and right) of reconstructed proprioception signals (top) and exemplar visual predictions (bottom), obtained by decoding the forecasted self latent space. See text for further explanation.

Formally, letting ${\hat{z}}^{s}(t)$ denote the predicted latent state, the dynamics of the neural-ODE are defined as:

(2.6)dz^s(t)dt=h(z^s(t),a(t)|θpred)(2.7)z^s(t0)=zs(t0),

where h is a neural network—a standard MLP in our implementation—parametrized by $\theta^{\mathrm{pred}}$, and the caret superscript $\hat{\cdot }$ makes explicit the difference between the modelled and target latent states. The loss function used to train the predictive component is:

(2.8) $$\begin{array}{r} L_{pred}=\sum_{i=1}^{T}\|{\hat{z}}^{s}(t_{i})-z^{s}(t_{i}){\|}_{2}^{2}+\sum_{i=1}^{T}||s(t_{i})-D^{p,s}\left({\hat{z}}^{s}(t_{i})\right)|{|}_{2}^{2}=L^{z}+L^{p}. \end{array}$$

In equation (2.8), the first term aligns the latent trajectory predicted by the neural-ODE with the latent activities produced by the correlation layer, while the second term penalizes errors in reconstructing proprioception. During predictive training, we use the latent space consistency loss $L^{z}$ in place of a visual reconstruction loss, thereby avoiding repeated and costly decoding passes. In all settings, unless otherwise stated, neural-ODE integration is performed using a second-order Runge–Kutta scheme with discretization step Δt=0.1. To assess the importance of using an explicit numerical integrator and a neural-ODE formulation, we also evaluate an autoregressive MLP baseline that predicts the latent state forward in time directly, without any integration method. Further details of variant models and their computational costs are provided in §2d and in the supplementary material.

#### (iii) Results

In figure 4, we show how the mean squared error (MSE) of the proprioceptive prediction error (panel b) and the pixel-wise error rate (1–accuracy) (panel c) depend on the temporal horizon T (equation (2.8)) and on whether numerical integration is used. The reported metrics are averaged over 100 step-ahead predictions. Overall, the errors decrease as the model is trained to predict longer trajectories, and incorporating an integration scheme yields consistently better performance. These results highlight the importance of treating prediction as learning a dynamical process over extended horizons, rather than as a one-step forecasting problem. Figure 4d shows how the same measures (MSE of the prediction error and pixel-wise error rate) vary as a function of prediction horizon for a neural-ODE model, with explicit integration, trained with T=50. As expected, error accumulates as the predictive layer rolls the latent state further into the future; nevertheless, pixel-level accuracy remains close to 90% even 100 steps ahead. In other words, despite the accumulation of error, the model is able to generate plausible future trajectories.

Figure 4c presents two examples (left and right) of reconstructed proprioceptive information for three representative variables with a range of dynamic variation. Targets are shown as dots and predictions as solid lines together with the corresponding reconstructed visual inputs at representative time intervals (bottom images). Predictions are reasonably accurate for the first 20 time steps and then begin to show underestimation errors while typically in the right direction. However, even at longer prediction horizons, the images preserve a coherent representation of the body and arms, with only finer details gradually deteriorating. Note that we omit the world latent from this analysis because its dynamics are exogenous to the agent and not action-conditioned. Learning them would require interaction-aware and stochastic dynamics using, for instance, a neural stochastic differential equation (neural-SDE) model [50,51]; we leave this to future work.

### (d) Scaffolding

#### (i) Overview/methods

Our architecture and learning procedures are deliberately modular—the model combines heterogeneous adaptive components with complementary cost terms, and optimization is performed in multiple stages that reflect an overall layered structure (figure 1).

In an earlier paper, Wilson & Prescott [43] defined *scaffolding* such that, given two processes, one with faster (earlier) dynamics, B, can serve as a scaffold for the organization of a slower (later) process, A, if the functional consequences of process B constrain A, such that some product of process A may be improved. These authors further suggested that scaffolding can occur without direct interaction between the two processes—all that is required is for process A to be measurably impacted by process B in a positive way.

In our architecture, we implement two forms of scaffolding motivated by our aim to understand the role of scaffolding in human development (as considered further in the Discussion). First, we pretrain the self pathway of the correlation layer (layer 0), while suppressing the world pathway, before simultaneously training both. This biases the model to find a way to reconstruct the visual scene using proprioceptive input rather than simply building a compressed representation of the scene. This is an example of scaffolding as the pretraining of the self pathway scaffolds learning in the world pathway even though they interact only directly (they share the same error signal and spatial mask). Second, we fix the encoders/decoders of the correlation layer prior to training the layer 1 predictive model. This stands as a more direct form of scaffolding as the self latent representation generated in layer 0 is provided as input to layer 1. Note that these two forms of scaffolding are similar in that both simplify the learning problem for the other part of the system. In the layer 0 case, this is by allowing the world pathway to focus on the non-self; in the layer 1 case, this is by providing as input a latent representation that is useful for learning how the self will evolve in time.

To test the utility of scaffolding, we next compare this *standard architecture* against variants in which scaffolding is removed or altered. From a machine learning perspective, it is also important to understand how scaffolding impacts on optimization. We therefore also test additional variants to better understand the behaviour of the scaffolded model as compared with end-to-end *backpropagation through time* (BPTT). End-to-end backpropagation is the standard approach for training deep neural networks as it enables gradients from the final objective to be propagated through all intermediate computations and, in recurrent settings, through time, thereby updating all parameters jointly to directly minimize loss. Although we expect BPTT to have high accuracy, one of the benefits of the standard architecture is that it may be computationally more efficient than other variants particularly those that update all parameters throughout training. We explore this further below and in the supplementary material.

Here, we explore five variants as defined below. For completeness, we also investigate a number of further variants in the supplementary material. All variants use numerical integration in the predictive layer and are trained with a time horizon of T = 50 until convergence. With the exception of variant (i), all variants also employ the self-pretraining phase.

(i) *W/o layer 0 scaffolding*. We remove the self-pretraining of the correlation layer and train all layer 0 pathways jointly from scratch. This directly tests the utility of the first form of scaffolding described above.
(ii) *Continuous training*. We jointly optimize both layer 0 and layer 1 throughout the whole training process. This tests whether there is a benefit to training level 0 to convergence before training level 1, as compared to training both layers from the outset. In other words, this tests the utility of the second form of scaffolding described above.
(iii) *End-to-end BPTT*. We jointly optimize both layers and allow prediction errors to flow through the entire architecture so that the correlation layer parameters (including encoders and decoders) are updated using gradients from both equations (2.5) and (2.8). We expect this variant to have good performance but to be computationally costly.
(iv) *W/o proprioception*. The standard architecture but with both layers trained without proprioceptive input though still trying to predict future proprioceptive states (i.e. retaining the loss term $L^{p}$). This condition tests whether visual information by itself is sufficient to induce a self–world distinction (for instance, through temporal correlation of moving body parts) and predict future self-states.
(v) *End-to-end backpropagation through time without proprioception (BPTT, w/o p)*. We jointly optimize both layers using backpropagation, as in variant (iii), but driving the system without proprioceptive input as in variant (iv). This condition again tests whether visual input is sufficient to induce a self–world distinction but this time under end-to-end optimization using backpropagation.

We should point out that it is also possible to continue to optimize layer 0 during layer 1 training, rather than freezing the layer 0 weights in the second training phase. This could further test whether having a stationary level 0 is beneficial to training level 1 (as compared to variant (ii) where both layers are trained from the outset). In practice, however, since layer 0 is trained to convergence during the initial phase, the weights would not change further during training of layer 1. In other words, this version will have a similar outcome to the standard model (although this would not be the case if the training data were to change).

#### (ii) Results

In figure 5, panel (a) shows exemplar image reconstructions from the self and world latent spaces for all five variants. Panels (b) and (c) report the standard model's soft Dice reconstruction scores and proprioceptive prediction errors, respectively, under different input regimes—*p*: proprioception only, *v*: vision only and *p+v*: a balanced mixture. The final two panels show the corresponding differences, post-convergence, between each variant and the standard baseline—panel (d) in terms of soft Dice scores and panel (e) for proprioceptive prediction errors. In these plots, self performance is reported at two horizons: t=0 (lighter), which evaluates correlation-layer separability, and t=50 (darker), which probes the predictive capacity of layer 1. In both plots, bars that extend to the left of the midline show outcomes that are worse than the standard model, while bars that extend to the right show better outcomes. Note that the soft Dice score for world reconstruction is provided for t=0 only since we are not attempting to forecast future world states (§2c).

**Figure 5.**
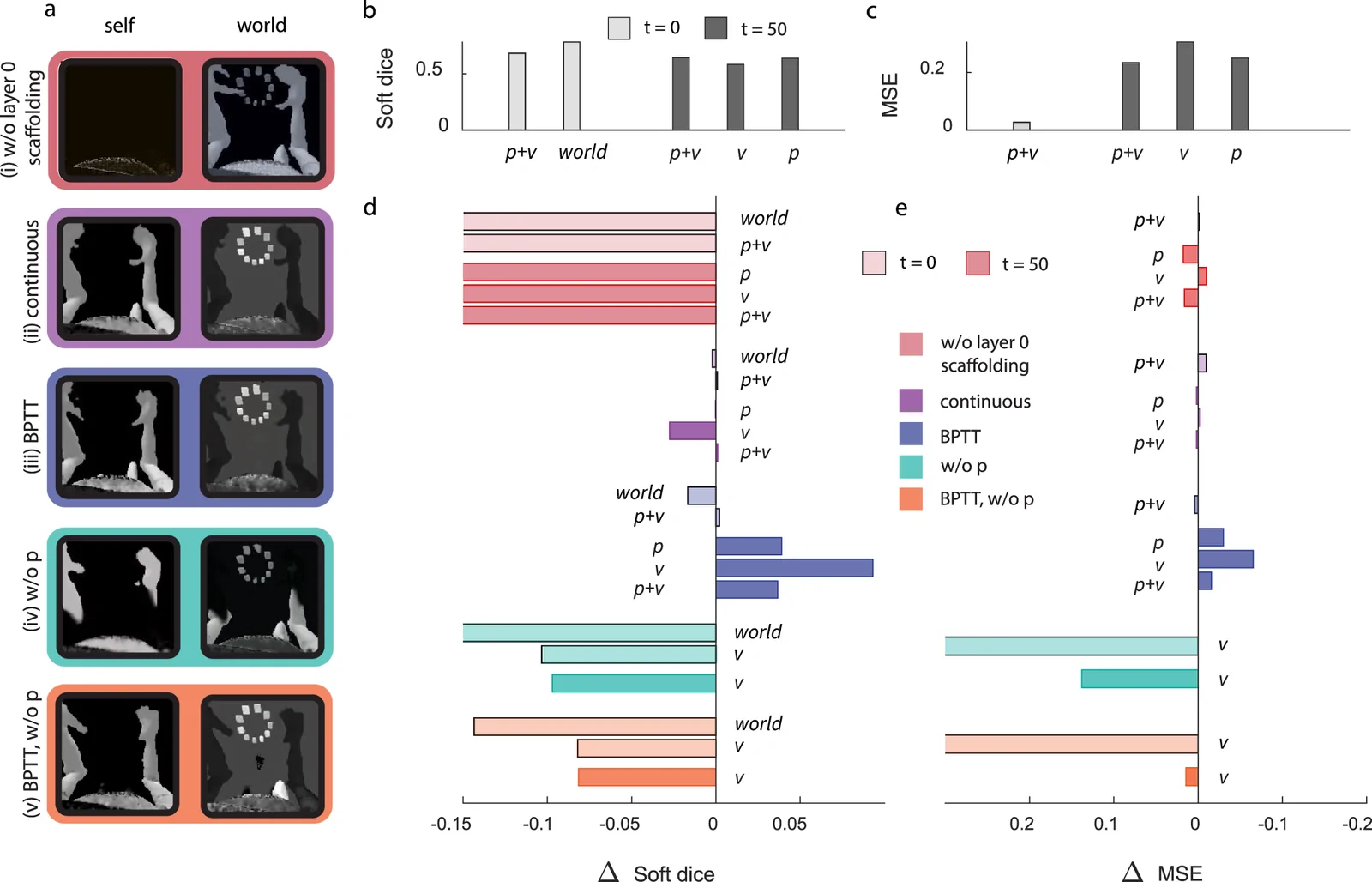
Results for model variants. (a) Example reconstructions (see figure 3d legend for description of elements), from top to bottom: w/o proprioception, BPTT w/o proprioception, continuous training, BPTT. (b) In lighter grey, self and world soft Dice for the reconstruction at the current time step (t=0) and the standard model. In darker colours, self soft Dice when the system is driven by proprioception (*p*), vision (*v*) or a mixture of the two for predicting 50 steps in the future (t=50). (c) Same as (b), but for the proprioception reconstruction error. (d) Accuracy differences with respect to the baseline standard model for the different settings explored (right is better). For variants (iv) and (v), trained without proprioception, we report performance under vision-only control, since the remaining settings require proprioceptive input. (e) Analogous to (d), but for the differences in the reconstruction errors.

Compared to the standard model, the five variants behave as follows:

(i) *W/o layer 0 scaffolding*. Removing the level 0 pretraining causes the network to reconstruct the entire scene almost exclusively through the *world* pathway (panel a(i)). Intuitively, while the self pathway must learn cross-modal correlations and reconstruct both proprioception and vision, the world pathway can more rapidly discover a purely visual encoder–decoder (i.e. by performing a form of compression) that therefore dominates early training. The resulting model is rather crude and performs poorly on visual reconstruction (panel d), but the architecture is able to construct a self latent that is sufficient to predict future proprioceptive states (panel e).
(ii) *Continuous training*. This variant achieves similar MSE to the standard model (panel e), but exhibits reduced soft Dice when the system is driven by vision alone (panel d). A likely explanation is that, in this setting, the predictive layer is trained against targets produced by the correlation layer that continue to change during optimization. This can destabilize learning and increase susceptibility to suboptimal local minima. We further note that this approach is less computationally efficient than the standard model (see below).
(iii) *BPTT*. As expected, end-to-end optimization improves both soft Dice (panel d) and prediction MSE (panel e), but at a substantial computational cost since it requires BPTT and through the full architecture (see below).
(iv) *W/o proprioception*. Removing proprioception prevents the model from establishing a robust self–world distinction, leading to poor self and world reconstruction (panel a(iv)) compared to the standard architecture. The soft Dice scores are much lower (panel d) and MSE scores much higher (panel e) for all tests.
(v) *BPTT*, *w/o p*. This variant has the same available information as variant (iv), which fails dramatically, but achieves marginally better performance. Indeed, it does surprisingly well at predicting future proprioceptive states (panel e). Overall, however, it fails to provide a satisfactory VSWD as indicated by the poor self–world separation in the image reconstructions (panel a(v)) and low soft Dice scores (panel d).

Overall, we observe that the staged training approach of the standard model performs well in these comparisons. Compared to variant (i), which lacks pretraining, it develops a reliable VSWD. Performance is similar to variant (ii) that allows joint optimization of both layers throughout the training process, and even marginally better on vision-only reconstruction of future self-states. Removal of the proprioceptive input (variant (iv)) demonstrates that the performance of the model leverages these internal signals in order to distinguish self from world and predict how the self will evolve.

As anticipated, end-to-end BPTT is able to improve on the results obtained with the layered architecture; however, this comes at some cost. By keeping the layer 0 encoders and decoders frozen during layer 1 training, the layered model obtains substantial efficiency savings—reducing the number of multiply–accumulate operations by around 98% compared to end-to-end BPTT. Indeed, we see substantial savings even in the continuous training setting (see the supplementary material for further explanation and detailed estimates). This performance improvement is achieved through the separation of timescales as predicted by Wilson & Prescott [43], with faster processes providing latent decompositions that scaffold acquisition of the slower predictive dynamics.

## 3. Discussion

With regard to the origins of the self in body exploration in early infancy, the developmental psychologist Phillipe Rochat wrote:

Self-produced action causes the infant to have overlapping experiences through his other senses. […] This experience specifies the body as differentiated from other objects in the environment. When my hand crosses my visual field, for example, I perceive that it is my hand and not someone else's, because I see it and feel it proprioceptively moving at exactly the same time and by a commensurate amount [5, p. 37].

In this contribution, we have investigated Rochat's proposal for the differentiation of self from world in infancy using a synthetic approach. Specifically, we have shown that a correlation-learning system can decompose the visual field of a simulated robot into self and non-self (figure 3). If proprioceptive signals are considered to already be part of the self because they originate from inside the body [5], then this learning system extends the self—in relation to an anoetic sense of body ownership—to parts of the visual world.

We have further shown that this minimal self–world distinction can usefully scaffold learning of continuous dynamical system models (neural-ODEs) that predict the future evolution of both proprioceptive and visual self-states (figure 4). Here, we have learned to predict the sensory outcomes of randomly generated ‘babbling’ actions; however, this predictive capacity should also be extendable to provide control signals for intentional, goal-directed actions (see below). Our model therefore aligns with theoretical discussions in which a pre-reflective sense of agency is seen as arising from congruence between the predicted sensory signals arising from voluntary action and the flow of reafferent sensory signals [26,45].

We next discuss our findings in relation to wider research on: (i) the minimal self in robotics, (ii) machine learning for multi-modal perception, (iii) the neural substrates for the human self–world distinction and its emergence during development, (iv) the role of scaffolding in the development of the human cognitive architecture, and (v) the future extension of the model towards noetic and autonoetic aspects of self.

### (a) Cognitive neurorobotics approaches to understanding the minimal self

Within the broader field of synthetic science [52], the current work can be regarded as following a *cognitive neurorobotics* methodology that uses robot experiments to explore potential mechanisms underlying embodied cognition [53,54]. Rather than attempting to capture the detailed behaviour of neural circuits, this approach focuses on exploring high-level algorithmic principles and on aligning system dynamics and behaviour with findings from neuroscience and psychology; as such, it complements computational neuroscience modelling and neurorobotics approaches at lower levels of analysis [54]. By taking inspiration from the trajectory of human cognitive development, our study also aligns with wider research in the field of *developmental robotics* [55]. Here, we briefly review some related work that has taken a similar approach to understanding the self–world distinction and sense of agency through robotics. (For wider discussions of robotic models of self, see [14,22,56,57].)

In a pioneering study of robot sense of self, Tani [58] explored a layered architecture in which a recurrent network was trained to predict the sequence of perceptual outputs of an associative network controlling a vision-based mobile robot in a navigation task. Tani [58] suggested that a form of self emerges in the dynamics of the predictive network when its predictions diverge from the outputs of the lower-level controller. Tani & White [53] refined this proposal suggesting that a form of implicit self is already present when the robot correctly predicts its future sensory states (as in the definition of pre-reflective agency above), but that when this synchrony breaks down, and effort is required to refocus the system, the distinction between self and world is brought into consciousness. This proposal suggests one way in which the current architecture could be developed towards a noetic (reflective) sense of self, through monitoring of whether anoetic systems are achieving intended goals (see further discussion below).

An early study of robot self-recognition, by Michel *et al.* [59], used the expected time delay between action initiation and observed arm movement to judge whether visual motion was self-generated. Saegusa *et al.* [60] subsequently showed that correlation learning can be used to allow a motor babbling robot to visually recognise its own body parts. This was further demonstrated by Demirel *et al.* [61] who used correlation-based learning of the relationship between robot arm velocities and tracked visual tags. Bechtle *et al.* [62] trained a forward model to predict the visual consequences of a humanoid robot's own movements and used this to attenuate sensory signals arising from the robot's own body. These authors related the behaviour of their model system to a sense of agency and also argued that suppressing self-related visual signals could assist the robot in acquiring object permanence [3]. Lanillos *et al.* [63–65] have developed various models of robot self-perception based on predictive learning frameworks. These included a visual self–other distinction, generated by using proprioceptive signals to predict visual input, which allowed a robot to recognize its own mirror reflection [65]. Our model builds on these previous approaches by including both correlational and predictive learning into one architecture. Specifically, our correlation layer maps heterogeneous inputs (vision and proprioception) into a compact, well-posed latent space upon which predictive learning systems can be defined that forecast self-related states. This extends previous cognitive neurorobotics models by showing how a layered model can emulate aspects of the developmental timeline for sense of agency seen in human infants (see further below).

### (b) Machine learning for multi-modal perception

The design of our model is also inspired by multi-modal state representation learning in robotics. For example, the proposed correlation layer is closely related to the variational autoencoder formulation of Meo & Lanillos [66]. This approach learns a joint latent from multiple sensors and enables cross-modal reconstruction under partial observability. Our architecture departs from this framework in three key ways. First, we explicitly factor the latent into a *self* component $z^{s}$ and a *world* component $z^{w}$, which (together) support information preservation while yielding an interpretable separation between agent and environment. Second, we endow the latent space with continuous-time dynamics via a neural-ODE driven by intended actions. This provides accurate prediction of future self-states through multi-step forecasting in latent space. Third, the presence of an explicit world representation, and of the dynamical prior, leads us to different architectural choices and loss terms from multi-modal autoencoder baselines. This connects our work to a growing literature in computer vision that has employed dynamical models to create image/video latent spaces. This research includes combining foundational neural-ODEs with latent ODEs [67,68], constructing second-order/physics-informed latent models [69,70] and neurally inspired or continuous-time video generation models [71,72]. These approaches have collectively demonstrated improved long-horizon rollout and more temporally consistent prediction compared to earlier work.

Predicting the future consequences of an agent's actions is closely related to the idea of using forward models in model-based reinforcement learning [73,74]. In this context, our architecture provides a simple self-supervised route to a self–world decomposition, which could help to structure learned world models by separating controllable (agent-related) from exogenous (environment-related) factors. Although speculative, such a factorization has clear potential advantages, including more modular reuse of predictive components across tasks and improved generalization when the agent or the environment changes independently.

### (c) The layered brain architecture for self–world discrimination

Panksepp [11,40] described a ‘primal self’ as arising from the convergence of emotional (evaluative), sensory and motor schemata with a body map and a core motivational arbitrator situated in subcortical regions of the brain. Key structures relevant to this include the periaqueductal grey (PAG), the hypothalamus and the medial reticular formation, all of which are important motivational and emotional structures [11,75], and the superior colliculus (SC) a critical site for multi-sensory convergence that is also involved in regulating head, eye and body movements [76,77]. All of these structures are appropriately connected to each other and to motor and sensory systems before birth and are operational in the human newborn. In addition, insular cortex, which is a key site for the convergence of exteroceptive and interoceptive signals, is likely to be involved in the early emergence of the self–world distinction and could encode an initial distinction between self and non-self based on interoception and self-touch [78,79]. Insular is the first cortical area to appear in the fetus and is considered to be both phylogenetically old and early maturing [80,81]. In figure 6, we highlight these structures, and particularly the SC and insular, as being potentially involved in a minimal, anoetic sense of self, and analogize them with layer 0 of our robot cognitive architecture (the correlation-learning layer). This proposal aligns with Rochat [5] and Rochat & Striano's [31] account of the emergence of a VSWD during early infancy that builds on a simpler non-visual distinction based on self-touch already present at birth. We next explore this neurodevelopmental proposal in more detail.

**Figure 6.**
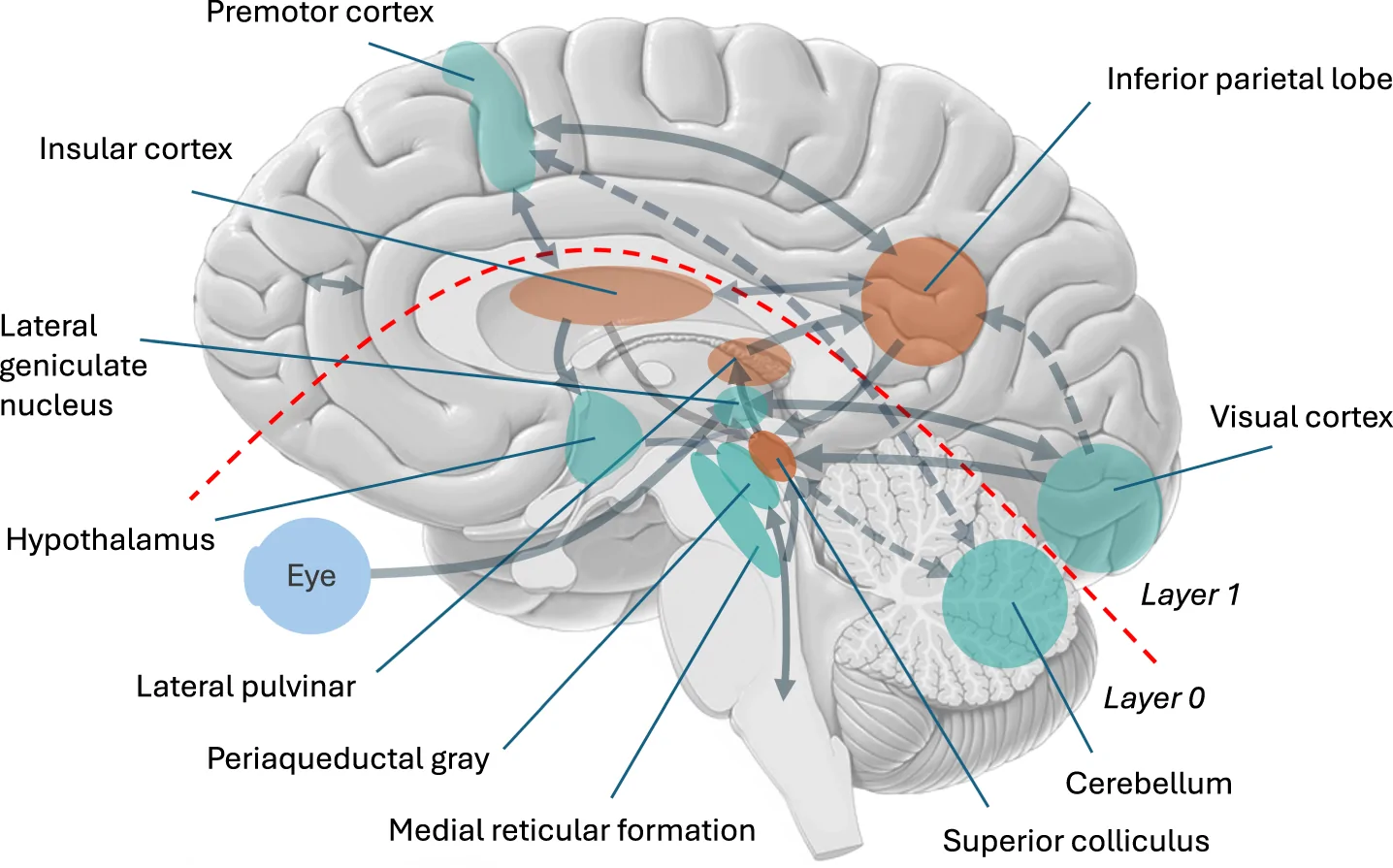
Some areas of the brain likely to be involved in the VSWD shown here above a sagittal section of the human brain. Key structures are highlighted in orange; other structures that process or relay signals relevant to a VSWD are highlighted in turquoise. Thick solid lines show direct connections; double arrowheads indicate that these are reciprocal; thick dashed lines show indirect connections. Only a subset of relevant structures and pathways are shown. Note that the insular cortex lies on an inner fold of the cortical sheet but is shown here overlying the sub-cortex. Layer 0 identifies a group of largely subcortical structures (but including insular) that are well-developed in the neonatal brain and could support an early form of VSWD similar to the correlation-based model in figure 3. We highlight the SC which receives direct input from the retina, and from multiple subcortical and cortical structures, and includes both a visual spatial map and activity related to proprioception. Layer 1 identifies later-developing cortical areas that could be involved in acquiring a more versatile form of VSWD similar to our predictive layer (figure 4). Of particular interest is the inferior parietal lobe (IPL), which is active during adult studies of agency and receives input from the SC via the lateral pulvinar and from insular cortex. Cerebellum is an important structure for learning to predict sensory signals; it may play an integrative role across the two layers. See text for further information.

As shown in figure 6, the SC receives direct input from the retina alongside indirect input from auditory and somatosensory systems and a wide range of subcortical and cortical structures. SC is both a sensory and motor structure and is often studied in relation to orienting to salient stimuli and the control of spatial attention [82]. Indeed, the superficial layers of the SC are well-known as encoding a head-centred representation of the world that aligns visual, auditory and tactile inputs [83,84], while the deeper layers act to generate eye and head movements to fixate or track objects of interest [76]. The SC is also known to provide corollary discharge signals for eye movements that help to stabilize visual perception [85]. However, while less studied, the SC and equivalent midbrain structures in non-mammalian vertebrates (the tectum) also play a wider role in movement generation (i.e. beyond orienting), including in reaching [86] and in whole body movement [87,88]. Interestingly, in primates, the deeper layers of the SC have been found to include cell populations that encode arm movements in gaze-related coordinates [89]. Other populations in SC, and the underlying PAG, show neural activity that correlates with shoulder, arm and trunk electromyography recordings during reaching [90], i.e. putative proprioceptive signals. Importantly, the SC does much more than simply provide a topographic map of the visual world. For instance, it is also involved in determining the salience of any objects in view with regard to future action [77,82]; indeed, in young infants, it is the primary site for this [91]. SC intrinsic circuitry has been modelled as implementing forms of correlation-based learning (e.g. [92,93]). Thus, in principle, SC seems a plausible site in the neonate brain for the acquisition of a VSWD, similar to that generated by layer 0 of our model, that could provide the developing infant with the capacity to identify areas of her visual world as belonging to self or world.

Cortical systems involved in visual processing mature rapidly during the first few month as they come to dominate the subcortical visual system [48,91]. There is evidence of function in visual cortical areas at birth [81], but the wider system is known to be incomplete and weakly integrated [91,94]. The brain expands in size during the first year, with cortical grey matter more than doubling, driven by the proliferation of intracortical synapses, myelination and increasing functional connectivity [94–96]. During this time, different cortical systems mature progressively, steered by experience [94,97] and beginning with sensorimotor and sensory areas, with a more gradual emergence of networks that process signals generated by these primary domains [96,98,99]. This aligns with theoretical proposals that cortical functional organization can be described by a hierarchy of predictive models [100].

From around three to four months of age, the infant brain gains the capacity to anticipate the future over short time frames, for instance, making prospective saccades towards the likely future position of a moving object or anticipating where an object might reappear that has become occluded (object permanence) [101,102]. As previously noted, predictively anticipating the sensory consequences of one's own movement is a key aspect of human agency [26,45,103]. Of the various cortical systems likely to be involved in a predictive VSWD the *inferior parietal lobe* (IPL) may be especially critical. In human adults, this area has been found to (i) encode the goal of motor actions [104], (ii) have topographic maps of the visual field and of the body [105,106], (iii) enable an allocentric body model that can be flexibly applied in representing the self or in reasoning about the actions of others [107], (iv) encode self-related body information [108], and (v) be active in studies that measure sense of agency [109,110]. As shown in figure 6, the IPL receives movement-related input from areas such as premotor cortex [104], information about the visual scene via the dorsal visual stream [111] and information about visually detected body parts from the extrastriate body area (EBA) [112]. It also reciprocally connects with the insular cortex [113], which, as we have already noted, may be one of the first cortical structures to be involved in the experience of self. With regard to its early development, there is evidence of functional activity related to action recognition in associative regions of cortex, including in areas that will become the IPL, emerging from around four to seven months [114,115].

Interestingly, the IPL has strong two-way connections with SC. Direct projections from IPL to SC terminate in the intermediate and deep layers [116,117], while IPL receives an indirect, topographically organized projection from SC superficial layers via the lateral pulvinar [116]. Input signals arriving in the lateral intraparietal area, which lies in the intraparietal sulcus (adjacent to the IPL), are not thought to be solely oculomotor but to be concerned with visual processing more generally [116]. Signals from SC may also be relayed to IPL via pulvinar projections to extrastriatal areas including the EBA [118]. It therefore seems plausible that the construction of a predictive VSWD, resembling that in layer 1 of our model, could involve the IPL and that this could build on subcortical representations of the visual field. This is consistent with imaging studies of the rubber hand illusion [119] that have implicated the SC as part of a broader system involved in bodily self-awareness in human adults.

A further important structure is the (hindbrain) cerebellum, which interacts with premotor cortex [120] and the IPL [116] via dysnaptic pathways involving the pontine nuclei and the thalamus. Its role in predicting sensory signals, including those related to self-movement, means that it is likely to be integral to the acquisition of a predictive VSWD [110]. The cerebellum also interacts reciprocally with the SC, again via the pontine nuclei [82,121]. The cerebellum has been modelled as an adaptive filter performing decorrelation [122], as being involved in calibrating collicular maps [123] and in sensory noise cancellation based on efference copy signals [124] (itself a form of self–world distinction). Cerebellar and pontine development is protracted but spikes during the early postnatal months. Although it is unclear to what extent the cerebellum could be involved in self–world discrimination in the earliest phase of infancy, its rapid post-birth development and interactions with key midbrain and cortical systems suggest that it could play an increasing, and potentially integrative, role as development proceeds.

### (d) Scaffolded development in layered architectures

Our model implements two forms of staged learning both of which can be regarded as forms of scaffolding [43].

At level 0, during training of the correlation-based layer, we first focus on the *self* pathway and suppress training of the *world* pathway. We do this to ensure that the model learns to use proprioceptive signals to predict visual self-movement. Developmentally, this could be considered to be a form of attention (overt or covert), which prioritizes some forms of signal over others. Interestingly, the visual system of neonates is known to have very limited depth of field such that objects beyond 30 cm are poorly discriminated [48]. In learning terms, this could have the benefit of allowing the infant to focus on proximal stimuli such as her own hand movements (also human faces), without being distracted by more distal stimuli. Infants are also known to spend time visually fixating their own hands [5], an activity that has been noted in newborns [125] and peaks before the onset of targeted reaching [36], thus ‘self-structuring’ [126] the visual signals provided to the developing brain.

Layer 1 scaffolding involves delaying training of the predicted layer until layer 0 training is completed and fixing the weights of the lower layer. This illustrates a broader principle that may apply elsewhere in human development, which is the capacity of representations acquired early on to scaffold learning in later maturing networks. In Wilson & Prescott [43], we discuss this in relation to the examples of infant face processing and mammalian thermoregulation. For example, there is good evidence of an early-developing subcortical face detection system, in the SC, that scaffolds the emergence of cortically based face recognition capacity [5,91,97]. We suggest that a similar form of scaffolding could be taking place in the emergence of the self–world distinction, again with the SC scaffolding learning in cortical structures such as IPL. Scaffolding is also likely to be important between cortical subsystems that form predictive hierarchies.

In the mammalian brain, there is significant postnatal development in the SC, particularly with respect to multi-sensory integration, regulated, at least in part, by top-down innervation from maturing cortical systems [127]. As noted above, representations in SC may also be modified and refined by developing SC-cerebellar circuitry. While we have focused in this discussion on scaffolding of the cortex by the sub-cortex, in Wilson & Prescott [43] we also discuss scaffolding of subcortical systems, such as the SC, by the cortex, but over much longer, evolutionary timescales. In evolving from the pre-mammalian tectum, we can expect that the SC will have changed under evolutionary pressure to better support cortical function, for instance, in constructing representations of the sensory world that are well-suited to act in consort with more predictively oriented cortical modules. In our architecture, the third term in equation (2.5), which enforces a form of temporal continuity in the layer 0 latent space (see analyses in the supplementary material), could be a model of this kind of evolution-imposed constraint.

In our analysis of model variants, we showed that our layered architecture approach is up to 98% more efficient than a prevailing machine learning benchmark (end-to-end BPTT) while still effectively segmenting the visual world and predicting future self-states. There are substantial efficiency gains even with the continuous training variant (see the supplementary material). Brain development is known to be energetically costly, with the brain in childhood requiring a significantly larger share of energy resource than in adults [128]. Indeed, the metabolic cost of brain development is considered to be an explanatory factor in the slow and protracted nature of childhood growth in humans compared to other mammals [128]. As training of AI models consumes an increasing share of the world's resources, we may be able to learn from the layered architecture of the human brain how to build and train very large network models more efficiently.

### (e) Beyond the anoetic self

We envisage extending our synthetic model to include further components and tiers with the goal of capturing wider aspects of the anoetic self and, beyond that, of the noetic and autonoetic self. Here, we briefly consider some paths forward.

#### (i) Visually guided sensorimotor behaviour

As previously noted, hand and arm movements in early infancy are largely exploratory though they can be accompanied by reflexive grasping when an object touches the palm of the hand [34,37]. Intentional movements towards visual targets emerge from around age three months [35] and goal-oriented reach and grasp behaviour from around five to seven months [35,36]. This development sequence has already inspired a number of developmental robotics approaches (e.g. [129,130]). We foresee extending the current model along the following lines.

In order to predict future self-states, layer 1 of our architecture acquires a model that is functionally similar to inverse kinematics; however, to construct this, we need targeted movement. We currently use an engineered PID controller to generate babbling movements that bring body parts into the visual field. A more biomimetic approach, based on studies of spontaneous movement in the fetus [34], would involve generating pre-reaching movements using combinations of muscle/motor synergies similar to those generated in the brainstem and spinal cord [131,132]. Such synergy patterns could themselves form the target for layer 1 training in place of the direct proprioceptive states used in the current model (see [133] for evidence that cortical systems select muscle synergies). Note that what we are proposing here can again be viewed as a form of scaffolding in which subcortical systems generate movement patterns and representations that are useful targets for cortical learning.

A further extension is to use our architecture to generate reaching behaviours towards visual targets as seen in three-month-old infants. To do this, we need to map reaching targets in visual coordinates to the (x, y, z) world coordinates used in our layer 1 model. This model can then be used to generate movement trajectories to target locations, for instance, to reach towards visually salient objects. It should be possible to acquire the required mapping either during motor babbling or by driving the layer 1 model with a distribution of sample actions and using the imagined visual consequences to generate end positions in head-centred visual space. This problem is relatively straightforward in our current robot model as there are no separate degrees of freedom for the head, and it would become trivial if we add the depth signal from the robot RGB-D camera. For a robot with a head/neck morphology more similar to humans, the spatial locations of target stimuli will need to be updated with respect to head movements (e.g. [134]). The extension to goal-directed reaching, as observed in five- to seven-month-old infants, will require the integration of sensorimotor control with motivation as discussed next.

#### (ii) Goal-directed behaviour

A further step will be to integrate evaluative and motivational systems into our architecture to build towards a more complete model of sense of agency and of goal-directed behaviour. In Prescott & Jimenez-Rodriguez [75], we analysed the mammalian brain architecture for motivation from a layered architecture perspective. This review identified several important subcortical substrates for motivated behaviour, including the medial reticular formation, the PAG, the hypothalamus and the basal ganglia. As noted above, these structures were also identified by Panksepp [11] as being implicated in the construction of a primal (anoetic) self equipped with ‘a primitive sort of intentionality (an automatized action readiness) and primitive forms of psychic coherence (global affective states of the brain)’ [11, p. 566]. Insular cortex is also considered to be a key forebrain structure for motivation, particularly in relation to homeostasis [78]. These structures likely contribute to the anoetic sense of agency, for instance, in relation to the experience of action authorship. Other cortical areas related to motivation are found in the medial and frontal cortical regions. These more slowly developing networks are usually associated with cognitive aspects of motivation, including representation of value, planning and monitoring, and are likely to be more involved in reflexive judgements about agency (see [75] and noetic self below). We expect to extend our layered architecture in the direction of goal-directed behaviour by building on some of our earlier neurorobotic models of relevant brain structures, including reticular formation [135], basal ganglia [136,137] and hypothalamus [138].

#### (iii) The self–other distinction

 We have focused here on the self–world distinction which is likely to be a pre-requisite for the self–other distinction [3–5], where other is understood as a social other. Theories of the development of a self–other distinction typically begin with the hypothesis that the human brain uses self-representations as a model for understanding the actions and intentions of others [56,139–141]. Indeed, some of the brain structures in figure 6, such as premotor cortex and the IPL, are often found to be active in experimental settings that test for this kind of self–other mapping [140]. The possibility of using a robot self-model to distinguish self from a human or robot other, or to reason about the actions of a social other, has already been investigated in a number of studies [22,56,65,141]. The current model will lend itself naturally to adding a further decomposition of the world that distinguishes socially important cues such as facial expressions and body movements as a means to inferring intentions. We have reported some prior work in this direction [142–144].

#### (iv) The noetic self

A layered systems view of self, which is also consistent with predictive processing approaches [100] and with the hierarchical nature of human cortical development [99], would conceive of the progression towards the noetic self as involving the addition of new processing layers. Research by Tani & White [53] (reviewed above) suggests one possibility, which is the emergence of a noetic self through processes that monitor the anoetic self and respond to errors. For instance, when expectations about the reafferent sensory signals generated by self-movement are violated (see also [26]). Candidate structures in human cortex include the anterior cingulate, which has been proposed as playing a role in cognitive control of more automatic processes [145] as part of a wider ‘salience’ network [146]. As additional processing layers are added, representations of self can be expected to be increasingly multi-modal, or amodal, to encode expectations (priors) over longer time spans and to be the subject of self-referential thought. In the human brain, this conceptual representation of self likely involves the *default mode network* (DMN) [147] as discussed next.

#### (v) The autonoetic self

The ability to reflect on the self as existing in time, the *autonoetic* self, is generally held to emerge more gradually in development and to follow other childhood milestones, such as language acquisition and autobiographical memory [13,14,148]. From around age two, children show early signs of an awareness of a personal past and future, for instance, by reporting on past events and through appropriate use of the past tense of activity verbs. However, the capacity to fuse together autobiographical memories and self-concept using narrative-like structures is typically not in evidence until the pre-school years [14,148]. In the brain, this likely involves higher-order networks in the superior parietal lobe, the posterior cingulate cortex and the hippocampus. These structures form part of the DMN which is active during self-reflective thinking and mental time travel [147,149]. We expect to investigate these ideas by integrating our work on the self–world distinction with research on event memory systems where we have used cognitive robotics approaches to test models, inspired by the hippocampal system, that reconstruct the past and anticipate the future in embodied robotic settings [14,144].

## 4. Conclusion

 We have argued that the human self–world distinction is acquired progressively and begins in an anoetic fashion (i.e. without self-awareness) with systems that learn to discriminate sensory signals emanating from (or generated by) the self, from those arising from the non-self. This distinction can guide attention, for example, towards the body for self-exploration, or away from it, to better understand the world. A basic distinction of this form can also support learning of more sophistical forms of self–world distinction, for instance, those that predict how the future will evolve. Here, we have demonstrated a layered control architecture for a simulated humanoid robot that implements these two forms of self–world distinction—one correlational, the other predictive—and have shown scaffolded (faster and better) learning of the predictive model if the correlation layer is already in place and provided as input.

We have framed this modelling approach in the context of human brain development and infant behaviour. Specifically, we have argued that early infancy might involve the acquisition of a non-predictive VSWD, analagous to our layer 0 network, largely in subcortical areas of the brain, and partly through the infant's observation of her own motor babbling. During the first year of life, and as the brain further develops, this could lead to the acquisition of a second form of VSWD, cortically based, predictive in nature and analogous to layer 1 in our model. This two-layered architecture, still essentially anoetic, demonstrates an important aspect of agency, specifically the ability to predict the future consequences of one's own actions. The analogy with our robotic model follows a cognitive neurorobotics methodology and is focused at the systems level rather than in terms of specific mechanisms. Nevertheless, we hope that this decomposition into pathways and layers, alongside the distinction between correlational and predictive learning systems, could prove helpful in understanding the emergence of the human self–world distinction, particularly with respect to the role of early-developing self subsystems in scaffolding later ones.

## Supplementary Material



## Data Availability

Our paper uses computational models. Code is accessible via a public Github repository at: http://github.com/LucaManneschi/Robot_Self_Sim.git. Supplementary material is available online [150].

## Theme

One contribution of 17 to a discussion meeting issue ‘From being to identity: analysis and synthesis of the self’.
