# Supplementary material for: Scaffolded acquisition of a robot self–world distinction in a layered architecture model of the developing self
Source: Philos Trans R Soc Lond B Biol Sci. 2026 Nov 10;381(1962):20250153. doi: 10.1098/rstb.2025.0153 (PMC13750418; doi:10.1098/rstb.2025.0153)
Supplement: Supplementary file 1 [file rstb.2025.0153.zip › rstb.2025.0153/rstb20250153_si_001.pdf]

PHILOSOPHICAL TRANSACTIONS B

royalsocietypublishing.org/journal/rstb

Research

Check for updates

Article submitted to journal

Subject Areas:

Computational Biology, Cognition, Neuroscience

Author for correspondence:

Tony J.Prescott

e-mail: t.j.prescott@sheffield.ac.uk

Scaffolded acquisition of a robot self-world distinction in a layered architecture model of the developing self—Supplementary material

Luca Manneschi[1], Alejandro Jimenez-Rodriguez[2], Daniel Camilleri[3] and Tony J.Prescott[1].

[1]School of Computer Science, the University of Sheffield, UK

[2]Department of Computing, Sheffield Hallam University, UK

[3]Kinematic Trees Ltd, Sheffield, UK

THE ROYAL SOCIETY PUBLISHING

© The Authors. Published by the Royal Society under the terms of the Creative Commons Attribution License http://creativecommons.org/licenses/by/4.0/, which permits unrestricted use, provided the original author and source are credited.

This supplement contains additional material concerning (a) the robot simulation and model architecture, and (b) further analyses of the layered architecture model and comparisons with model varian models with regard to computational cost and performance.

(a) Robot simulation and model architecture

We first provide additional details in relation to (i) accuracy measures for model performance, (ii) the specification of the correlation-learning layer, (iii) the specification of the predictive layer, and (iv) the training procedure for the standard model.

(i) Accuracy measures

To quantify how well each latent space captures *self* and *world* in the visual domain, we compare the decoder reconstructions against ground-truth segmentations using two complementary overlap metrics. *Pixel-level accuracy* yields a simple, discrete score by converting the model output into a binary mask via a top-k selection. On the other hand, the *soft Dice coefficient* [1] is computed from continuous reconstruction scores and provides a graded measure of overlap. In both cases we evaluate *self* and *world* separately, using the corresponding ground-truth masks and a class-specific choice of k.

We denote the reconstructed visual outputs of the self and world decoders at time t by $\mathbf{x}^{v,\mathrm{s}}(t)$ and $\mathbf{x}^{v,\mathrm{w}}(t)$, respectively, with $\mathbf{x}^{v,\cdot}(t) \in \mathbb{R}^{H\times W\times C}$, and we denote the ground-truth visual input by $\hat{\mathbf{x}}^{v}(t)$. From each reconstruction we form a per-pixel *intensity* map by averaging across channels:

$$\mathbf{a}^{c}_{ij}(t) = \frac{1}{C}\sum_{k=1}^{C} \mathbf{x}^{v,c}_{ijk}(t), \qquad c \in \{\mathrm{s},\mathrm{w}\}. \tag{0.1}$$

Let $\hat{\mathbf{m}}^{\mathrm{s}}(t), \hat{\mathbf{m}}^{\mathrm{w}}(t) \in \{0,1\}^{H\times W}$ denote the ground-truth binary segmentations for self and world. For each class $c \in \{\mathrm{s},\mathrm{w}\}$, we set

$$k_c = \frac{1}{T}\sum_{t=1}^{T}\sum_{i=1}^{H}\sum_{j=1}^{W} \hat{\mathbf{m}}^{c}_{ij}(t), \tag{0.2}$$

i.e., the average number of pixels assigned to class c in the ground truth (rounded to the nearest integer in practice). We then define $\mathcal{T}_{k_c}(\mathbf{a}^{c}(t))$ as the map that retains the k_c largest values of $\mathbf{a}^{c}(t)$ and sets all others to zero, and we binarise it to obtain the top-k_c mask

$$\mathbf{m}^{c}_{ij}(t) = \begin{cases} 1, & \text{if } \mathbf{a}^{c}_{ij}(t) \text{ is among the top-}k_c \text{ values of } \mathbf{a}^{c}(t), \\ 0, & \text{otherwise.} \end{cases} \tag{0.3}$$

Pixel-level accuracy. Pixel-level accuracy for class c is computed by comparing $\mathbf{m}^{c}(t)$ to the corresponding ground-truth segmentation $\hat{\mathbf{m}}^{c}(t)$:

$$\mathrm{Acc}^{c}(t) = \frac{1}{HW}\sum_{i=1}^{H}\sum_{j=1}^{W}\Big(1 - \big|\mathbf{m}^{c}_{ij}(t) - \hat{\mathbf{m}}^{c}_{ij}(t)\big|\Big). \tag{0.4}$$

Soft Dice. To obtain a continuous overlap measure, we compute the soft Dice coefficient on the *non-binarised* top-k_c intensities. Let

$$\tilde{\mathbf{a}}^{c}(t) = \mathcal{T}_{k_c}\big(\mathbf{a}^{c}(t)\big) \in \mathbb{R}^{H\times W}_{\geq 0}. \tag{0.5}$$

The soft Dice score for class c is

$$\mathrm{Dice}^{c}_{\mathrm{soft}}(t) = \frac{2\sum_{i,j}\tilde{\mathbf{a}}^{c}_{ij}(t)\,\hat{\mathbf{m}}^{c}_{ij}(t) + \epsilon}{\sum_{i,j}\tilde{\mathbf{a}}^{c}_{ij}(t) + \sum_{i,j}\hat{\mathbf{m}}^{c}_{ij}(t) + \epsilon}, \tag{0.6}$$

with a small $\epsilon > 0$ for numerical stability. We compute the metric separately for self ($c = \mathrm{s}$) and world ($c = \mathrm{w}$) using their respective ground-truth segmentations and class-specific k_c.

(ii) The correlation-learning layer

Following the formalism in the main text (section 2b), the visual encoders $E^{v,\mathrm{s}}$ and $E^{v,\mathrm{w}}$ are compact CNNs with a ResNet-style topology: four residual blocks with output channel counts $\{4,16,32,64\}$; unless otherwise stated, each block uses a 3×3 convolution, batch normalization, ReLU, and a residual skip (with downsampling on the first convolution of a block when the spatial resolution changes). The corresponding visual decoders $D^{v,\mathrm{s}}$ and $D^{v,\mathrm{w}}$ mirror the encoders with four transposed-convolution blocks that invert the encoder's downsampling pattern. The visual input at time t is augmented with a fourth channel that encodes inter-frame motion: letting x_t denote the RGB frame and $\mathrm{gray}(x_t)$ its grayscale version, we define the raw temporal difference $\Delta_t = \mathrm{gray}(x_t) - \mathrm{gray}(x_{t-1})$ and smooth it with an exponential moving average (EMA) $\tilde{\Delta}_t = \beta\,\tilde{\Delta}_{t-1} + (1-\beta)\,\Delta_t$ with $\beta \in [0,1)$; the smoothed map $\tilde{\Delta}_t$ is used as the fourth input channel ("flow"), providing a stable, parameter-free motion cue. For the proprioceptive input $\mathbf{s}(t) \in \mathbb{R}^{N_s}$, we use a linear projection $E^{p}(\mathbf{s}(t)) = \mathbf{W}\,\mathbf{s}(t)$, where $\mathbf{W} \in \mathbb{R}^{N_z\times N_s}$ is initialized so that its top $N_s \times N_s$ block equals the identity (all remaining rows initialized near zero), thereby biasing the self pathway with a near-identity transformation at the start of learning and leveraging the typically well-behaved dynamics of proprioceptive signals to seed a

predictable latent space; this constraint can be relaxed by using an MLP or even a recurrent neural network as the encoder, but we maintain a minimal and effective model for simplicity. The proprioceptive decoder $D^{p,s}$ is an MLP with two hidden layers and ReLU activations. This asymmetry between encoding and decoding for proprioception is designed to drive the latent space with information that closely resembles $\mathbf{s}(t)$ while allowing flexibility in the decoding stage, which we found important to map information coming from the visual input to the proprioception. Both latent spaces, $\mathbf{z}^{\mathrm{s}}(t)$ and $\mathbf{z}^{\mathrm{w}}(t)$, lie in $\mathbb{R}^{N_z}$ with $N_z = 100$ in our simulations. We define the self latent $\mathbf{z}^{\mathrm{s}}(t)$ as a linear combination of signals from the different modalities. Other fusion strategies—such as concatenation or attention-based fusion—are also viable; the latter is especially promising given advances in multimodal attention/transformers [2,3]. Here, however, we deliberately adopt the simplest implementation to isolate the effect of the architectural structure.

For optimization, we use Adam (Adaptive Moment Estimation) [4] with a learning rate of 10^{-3}, a batch size of 32, and trained all models for 60,000 iterations (10,000 iteration during stage 1 and 50,000 iterations during stage 2), which is beyond their convergence time.

(iii) The predictive layer

The predictive layer is a neural-ODE driven by an external signal $\mathbf{a}(t)$, defined as the desired change of position $\Delta(x, y, z)$, of the end effector (both hands) with respect to a randomly selected babbling target. The input to the model is updated each time the target position changes. Its vector field f_ψ is parameterized by an MLP with two hidden layers of width 200 and ReLU activations trained with the Adam optimizer on temporal trajectories of length $\mathrm{T} = 50$. During training, we sample a random starting time within each sequence of the dataset and minimize the predictive loss in Eq. 2.7. For simplicity, we do not reconstruct pixels; instead, we reconstruct the encoded latent representation produced by the correlation layer. While an additional image-reconstruction term could be included, the consistency between the correlation layer's latent space——already optimized to be decoded——and the evolution enforced by the predictive layer provides sufficient signal for effective learning, as evidenced by the decoded, evolved latent trajectories (see Fig. 4e in the Main text). Consequently, in this stage we freeze the encoders and decoders and train only the predictive neural-ODE, which reduces computational cost while preserving feasibility, since it already requires evolving trajectories for multiple steps in latent space. In this sense, the strategy already functions as a form of *scaffolding* that improves computational time and overall feasibility.

(iv) Training procedure

Training of the standard architecture proceeds in two phases. First, we train the correlation/encoding modules (including the visual decoder) and then *precompute* the latent trajectories $\mathbf{z}^{\mathrm{s}}(t)$ over the entire dataset. In the second phase, we train the neural-ODE (with activities $\hat{\mathbf{z}}^{\mathrm{s}}(t)$, see Main text) to predict the latent sequence by minimising

$$\mathcal{L}_z = \frac{1}{T}\sum_{t=1}^{T} \left\| \hat{\mathbf{z}}^{\mathrm{s}}(t) - \mathbf{z}^{\mathrm{s}}(t) \right\|_2^2.$$

in place of the visual reconstruction. Because the decoders have already been trained to reconstruct inputs from $\mathbf{z}^{\mathrm{s}}(t)$, this latent-space loss acts as a surrogate for the reconstruction objective and lets us *bypass decoding during predictive training*. Practically, the scaffolding allows us to train the recurrent model directly in the compact latent space, avoiding the heavier visual encoders/decoders and yielding substantial compute savings. In practice, we encode the dataset once to precompute the targets $\mathbf{z}^{\mathrm{s}}(t)$, and then train directly in latent space.

(b) Additional analyses

We provide further analyses of (i) the computational benefits of the layered architecture approach, (ii) the structured nature of the predictive layer latent space, (iii) the role of temporal regularisation in optimising training, (iv) the role of proprioception, and (v) learning curves for model variants.

(i) Computational benefits of the layered architecture approach

As a measure of compute cost we count *multiply–accumulate* operations (MACs) per sample. For common layers:

$$\text{Dense: } \mathrm{MAC} = n_{\text{in}}\, n_{\text{out}}, \qquad \text{Conv2D: } \mathrm{MAC} = C_{\text{out}}\, H_{\text{out}}\, W_{\text{out}}\, (C_{\text{in}}\, K_h\, K_w).$$

(Per-batch cost scales linearly with batch size; biases/BN/activations are neglected.)

Training cost models. Let NFE be the number of function evaluations of the ODE solver and f_θ the ODE MLP. For a sequence of length T,

$$\mathrm{MAC}_{\text{scaffold}} \approx T\,\mathrm{NFE} \sum_{\ell \in f_\theta} \mathrm{MAC}_\ell, \qquad \mathrm{MAC}_{\text{traditional}} \approx \underbrace{\sum_{\ell \in \text{enc}} \mathrm{MAC}_\ell}_{\text{encode once}} + T\,\mathrm{NFE} \sum_{\ell \in f_\theta} \mathrm{MAC}_\ell + \underbrace{L \sum_{\ell \in \text{dec}} \mathrm{MAC}_\ell}_{\text{decode } L \text{ times}}.$$

In the traditional (end-to-end) setting we train all modules *simultaneously*: we run the encoder once to obtain the initial latent state, evolve the latent trajectory for T steps with the neural-ODE, and then decode at a conservatively subsampled set of L time indices $\{t_1, \dots, t_L\} \subseteq \{1, \dots, T\}$. The objective replaces the latent-space consistency used above with the reconstruction loss,

$$\mathcal{L}_{\text{recon}} = \frac{1}{L} \sum_{l=1}^{L} \left\| \mathbf{x}^v(t_l) - \hat{\mathbf{x}}^v(t_l) \right\|_1.$$

Instantiating for our architecture. Visual input 128×128, channels $\{16, 32, 64\}$, 3×3 kernels, max-pool after the stem, projection head 65,536→200→100. The convolutional part contributes

$$\begin{aligned} &16 \cdot 128 \cdot 128 \cdot (4 \cdot 3 \cdot 3) \\ &\quad + 32 \cdot 64 \cdot 64 \cdot (16 \cdot 3 \cdot 3) + 32 \cdot 64 \cdot 64 \cdot (32 \cdot 3 \cdot 3) \\ &\quad + 64 \cdot 32 \cdot 32 \cdot (32 \cdot 3 \cdot 3) + 64 \cdot 32 \cdot 32 \cdot (64 \cdot 3 \cdot 3) \approx 1.23 \times 10^8 \text{ MACs}. \end{aligned}$$

The two residual 1×1 downsample paths add $\approx 4.2 \times 10^6$ MACs, and the projection MLP head to the latent space adds

$$65{,}536 \times 200 + 200 \times 100 \approx 1.3 \times 10^7 \text{ MACs}.$$

Hence the *visual encoder* totals

$$\sum_{\ell \in \text{enc}^v} \text{MAC}_\ell \approx (1.23 + 0.42 + 0.13) \times 10^8 \approx 1.40 \times 10^8 \text{ MACs}.$$

The proprioception MLP (linear encoder 100×100 and two-layer decoder $3 \times (100 \times 100)$) contributes

$$\sum_{\ell \in \text{enc}^p} \text{MAC}_\ell + \sum_{\ell \in \text{dec}^p} \text{MAC}_\ell \approx 4.0 \times 10^4 \text{ MACs}.$$

The neural–ODE field f_θ (MLP 114→200→200→100) requires

$$\sum_{\ell \in f_\theta} \text{MAC}_\ell = 114 \cdot 200 + 200 \cdot 200 + 200 \cdot 100 \approx 8.3 \times 10^4 \text{ MACs per FE}.$$

Totals and savings. For $T = 50$:

- **RK2** ($\text{NFE} = 2 \Rightarrow T\,\text{NFE} = 100$):

$$\text{MAC}_{\text{scaffold}} \approx 100 \times 8.3 \times 10^4 \approx 8.3 \times 10^6, \quad \text{MAC}_{\text{traditional}} \approx 1.40 \times 10^8 + 8.3 \times 10^6 + 2 \times 1.40 \times 10^8 \approx 4.28 \times 10^8.$$

In this regime, training in latent space (scaffolding) avoids repeated image decoding and yields a large reduction in training MACs (well beyond 5× under conservative settings). Even in settings where end–to–end optimisation of the full architecture may confer accuracy gains, the scaffolding regime remains valuable: it supplies a strong *initialisation*, reduces how often decoding is invoked during training, and thus preserves substantial computational savings (with optional end–to–end fine–tuning applied afterward). The separation we exploit is facilitated by static (non-recurrent) encoders and decoders. Nevertheless, analogous savings can still be obtained when these modules are recurrent, provided the predictive layer evolves on a slower timescale than upstream processing. In this separation of timescales, faster processes can furnish latent summaries that scaffold the slower dynamics, allowing decoding to be triggered only intermittently while retaining the computational benefits.

Finally, even in comparison to *continuous training* (see main text), where we do not backpropagate through the full architecture, but still optimise the encoders/decoders while training the predictive layer, the scaffolding scheme can offer computational advantages. In particular, the predictive layer can still be trained using precomputed latent trajectories, amortising the cost of encoding across many optimisation steps. In the continuous-training regime the encoders change while the predictive layer is being optimised, which would be inexpensive if both layers were trained on the same data (since a single forward pass through the encoders would supply latent targets for both). However, the predictive objective requires learning over temporal sequences, making the encoding stage a repeated bottleneck if latent trajectories must be recomputed at each optimisation step.

(ii) Structure of the neural-ODE latent space

First, we focus on the predictive layer (layer 1) and show that the learned neural-ODE vector field, $\mathbf{f}(\mathbf{x}(t), \mathbf{a}(t) \mid \boldsymbol{\theta}^{\text{p}})$, which governs the evolution of the latent state $\hat{\mathbf{z}}^{\text{s}}(t)$, is highly structured. In particular, it exhibits a spiral-like flow consistent with the robot's babbling dynamics. This is illustrated in Fig. S1 where panel (a) shows the estimated vector field in blue and an example latent trajectory in red, while panel (b) illustrates how the latent space represents one of the key dimensions—the distance of the arms to the body.

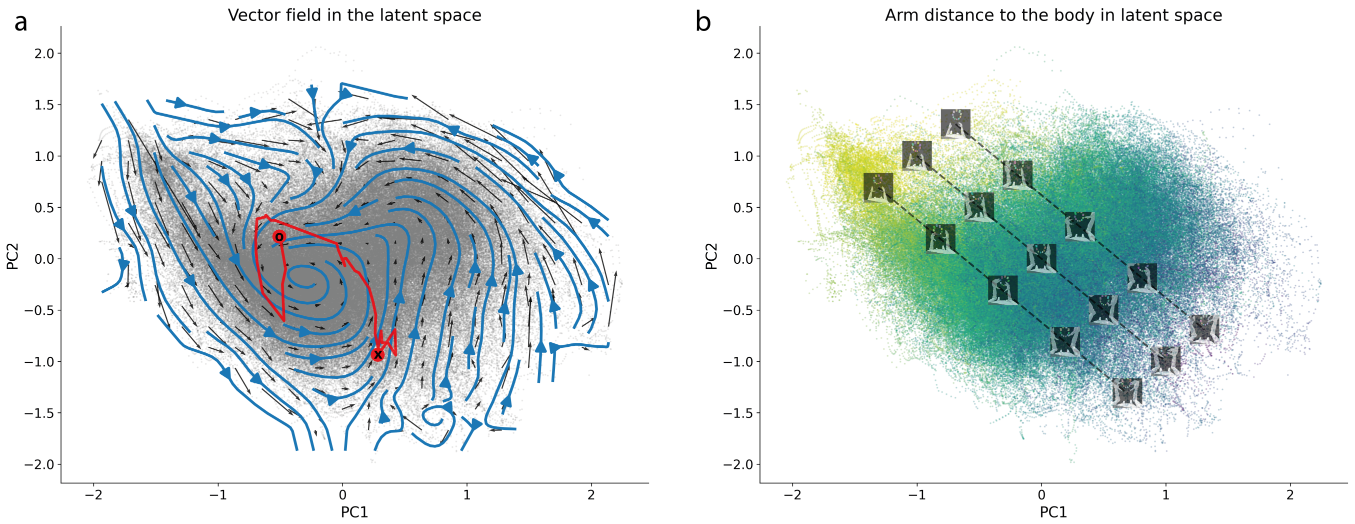

Figure S1. Structure of the latent space. (a) Visualisation of the first two principal components of the Neural-ODE vector field, showing how the predictive layer is learning a structured latent space. To visualise this behaviour in a low-dimensional (PCA) embedding, we estimate $\mathbf{f}$ at each location in PCA space by averaging the predicted derivatives over multiple trajectories that pass near that point, yielding a smooth vector-field representation. The blue lines correspond to the integrated vector field computed as the average direction of the trajectories in bins centred at the starting point of each black arrow. The red curve illustrates an example trajectory starting at 'x' and ending at 'o'. (b) Distance of the arms to the body mapped as a heatmap onto the latent space. Each latent vector is coloured by the distance of the associated elbow-pitch DOF and the shoulder joint. The yellow region shows hand positions that are far from the head. The blue region, frames in which the hand is close. Overlaid are example images from trajectories that pass trough a selected partition of the three parallel lines aligned with the gradient Plots were computed using the standard architecture trained to convergence with a T=50 time horizon.

(iii) The role of temporal regularisation

We next examine the role of the temporal regularisation term β in the loss function for the correlation-learning layer (layer 0) (see Eq. 2.5 in the main text). This parameter determines the extent to which we enforces temporal proximity across time within the latent space with higher values of β imposing a stronger constraint. In Fig. S2, panels (a)–(c) report the proprioceptive prediction error, pixel-level accuracy, and the normalised mean-squared error (NMSE) of latent forecasting as β varies for the standard model where regularisation is imposed only on the self latent space $\mathbf{z}^{s}(t)$. Although the effect of β is modest, performance suggests an optimal value with $\beta > 0$, indicating that temporal regularisation helps shape the latent space learned by the correlation layer to be more amenable to predictive modelling.

Interestingly, when world-related information is also provided as input to the neural-ODE (panels (d)–(f)), i.e. both self and world latent spaces $(\mathbf{z}^{s}(t), \mathbf{z}^{w}(t))$ are regularised, the value of β becomes more critical. (Note, this is variant of the model that we did *not* investigate in the main paper). For $\beta = 0$ the model performs close to chance level and exhibits unstable NMSE (panel (f)). Increasing β stabilises optimisation and yields satisfactory performance, likely because the regulariser now also constrains the world-related components that are harder to model deterministically. We emphasise that we are not forecasting the world state in this setting—doing so would require stochastic modelling—but these results highlight the importance of temporal regularisation when exogenous world information is injected into a deterministic predictive layer. Overall, the findings support the view that reliable deterministic predictability is primarily a property of the self, whereas incorporating world-related factors can undermine long-horizon prediction unless appropriately regularised.

(iv) The role of propriception

Next, we include two additional ablation settings to further analyse the architecture with respect to the role of proprioception. In these new conditions, termed *w/o p and* $\mathcal{L}^{p}$ and *BPTT, w/o p and* $\mathcal{L}^{p}$, we remove both the proprioceptive input *and* the proprioceptive reconstruction objective $\mathcal{L}^{p}$. The two cases differ in whether prediction errors are backpropagated from the predictive layer to the correlation layer. These new variants contrast with variants tested in the main paper where we removed the proprioceptive input but retained the loss term $\mathcal{L}^{p}$ therefore requiring the model to try to reconstruct future proprioceptive states. In other words, these new configurations remove all proprioceptive pathways and isolate the vision-only regime, allowing us to test whether predictability alone can support a self-world separation of the visual image.

As can be seen from Fig. S3, which should be viewed as expanding Fig. 5 in the main text, both settings perform poorly compared to the standard architecture. However, these settings do achieve slightly higher reconstruction accuracy than the corresponding variants (*w/o p*) and (*BPTT, w/o p*) from the main study. This can be explained by noting that when the model is asked to reconstruct proprioception from vision alone, without a reliable guiding signal (the proprioception), training may

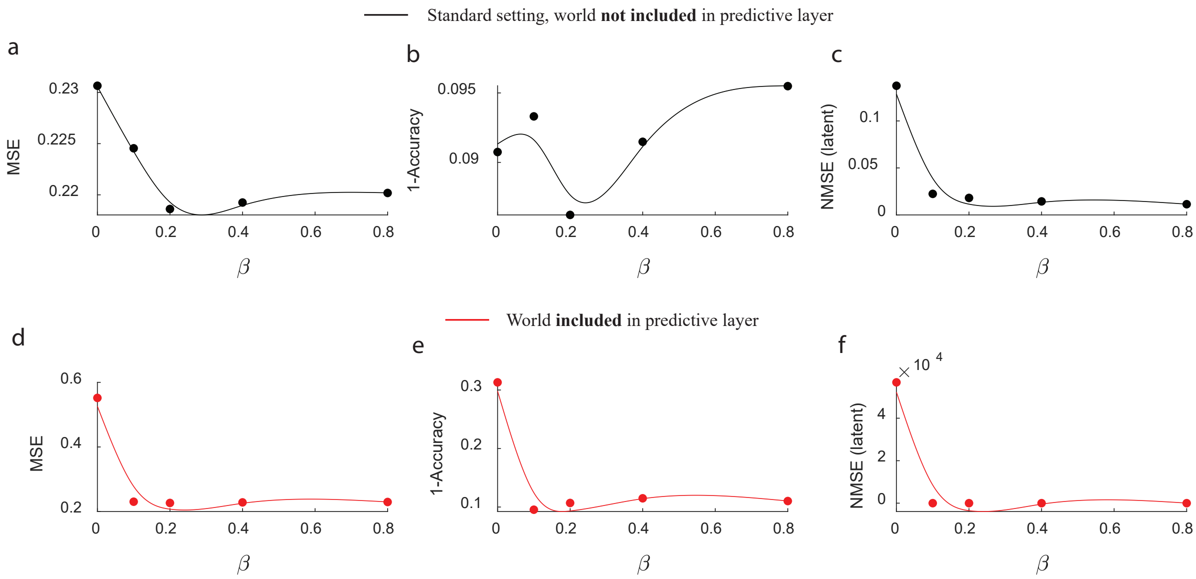

Figure S2. Role of the temporal regularisation term as its importance, defined via the scaling factor β, varies. Increasing β steers the latent space of the model toward smoother dynamics across time. (a,b,c) Performance for the standard setting, where the world-related information is not included as input to the predictive layer (the neural-ODE). (d,e,f) Performance when the world latent space provides additional input to the neural-ODE. In this case, the regularisation term is acting on **both** self and world latent representations. (a,d) Proprioceptive errors reconstruction for the two cases as β increases. (b,f) Pixel-level accuracy. (c,f) Normalised mean squared errors of the latent space reconstruction.

converge to suboptimal local minima, which in turn degrades the quality of the learned self-world separation.

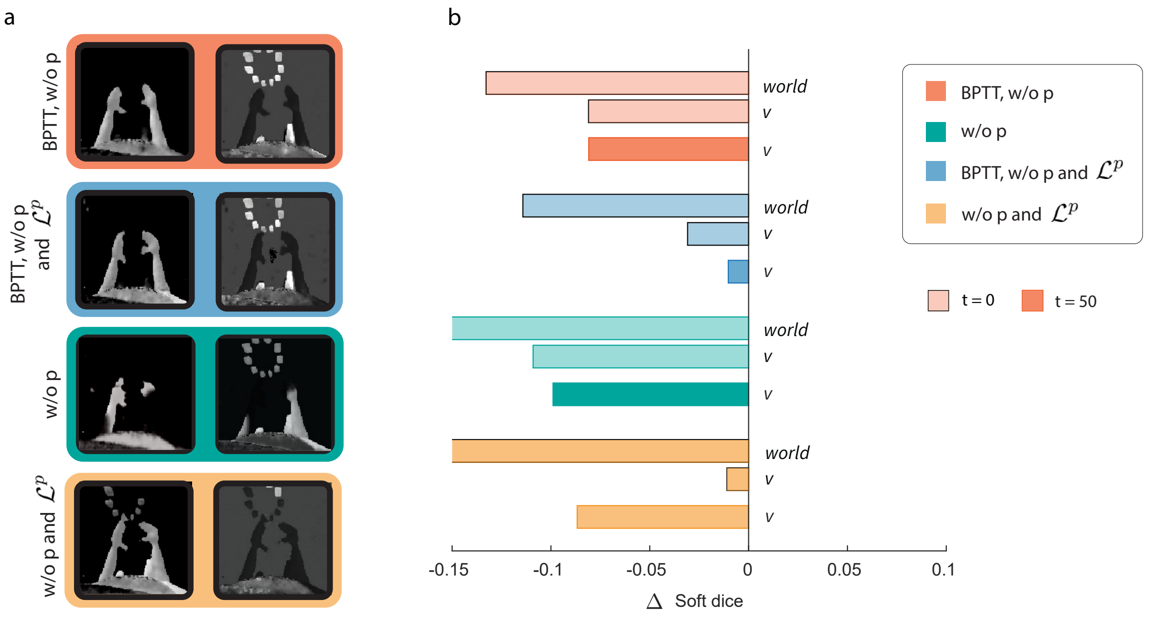

Figure S3. Results for two new model variants in which we remove both the proprioceptive input *and* the proprioceptive reconstruction loss. We refer to these new cases as *w/o p and* $\mathcal{L}^p$ and *BPTT, w/o p and* $\mathcal{L}^p$, which differ in whether backpropagation through time is applied across the full architecture. We reproduce the results for variants *iv* and *v* from Figure 5 in the main text comparison.

(v) Learning curves for model variants

Finally, Fig. S4 shows example training curves to illustrate the convergence behaviour and performance of several variants. Panels show (a) normalised mean squared error (NMSE) for the latent space prediction, and (b) NMSE for predictive forecasting over the

first 40,000 training iterations sampled at logarithmic intervals. Here, *w/o* $\mathcal{L}^p$ denotes the condition in which the proprioceptive loss term is removed during Layer 0 training, we also illustrate the variants *continuous training* (variant *ii* in the main text) and *without proprioception* (variant *iv*). Note that the standard model and continuous training variants train to near convergence, with standard architecture showing slightly faster/better learning of the latent space prediction.

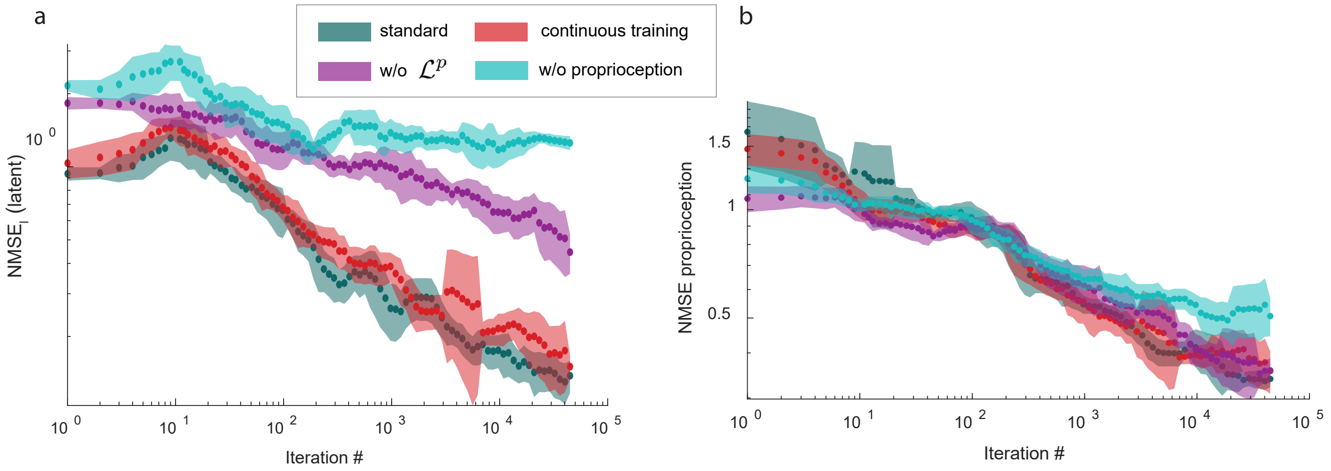

Figure S4. Learning curves for example variants, showing how the standard model leads to improved learning curves in comparison to the other cases (neglecting BPTT). (a) Normalised mean squared error for the latent space prediction. (b) NMSE for proprioception forecasting. Note that iteration number is plotted using a log scale.

References

1. Milletari F, Navab N, Ahmadi SA. 2016 V-net: Fully convolutional neural networks for volumetric medical image segmentation. In *2016 Fourth International Conference on 3D Vision (3DV)* pp. 565–571. IEEE.
2. Tsai YHH, Bai S, Liang PP, Kolter JZ, Morency LP, Salakhutdinov R. 2019 Multimodal transformer for unaligned multimodal language sequences. In *Proceedings of the conference. Association for computational linguistics. Meeting* vol. 2019 p. 6558.
3. Xue H, Huang Y, Liu B, Peng H, Fu J, Li H, Luo J. 2021 Probing inter-modality: Visual parsing with self-attention for vision-and-language pre-training. *Advances in Neural Information Processing Systems* **34**, 4514–4528.
4. Kingma DP, Ba J. 2017 Adam: A Method for Stochastic Optimization. *arXiv, cs.LG,* **1412.6980, https://arxiv.org/abs/1412.6980**.
